# Supplementary material for: Quantitative live cell imaging of a tauopathy model enables the identification of a polypharmacological drug candidate that restores physiological microtubule interaction
Source: Nat Commun. 2024 Feb 23;15:1679. doi: 10.1038/s41467-024-45851-6 (PMC10891143; doi:10.1038/s41467-024-45851-6)
Supplement: Supplementary file 1 — Supplementary Information [file 41467_2024_45851_MOESM1_ESM.pdf]

SUPPORTING INFORMATION

Quantitative live cell imaging of a tauopathy model enables the identification of a polypharmacological drug candidate that restores physiological microtubule interaction

Luca Pinzi[1, #], Christian Conze[2, #], Nicolo Bisi[2], Gabriele Dalla Torre[1, λ], Ahmed Soliman[2], Nanci Monteiro-Abreu[2], Nataliya I. Trushina[2], Andrea Krusenbaum[2], Maryam Khodaei Dolouei[2], Andrea Hellwig[3], Michael S. Christodoulou [1, 4, Ω], Daniele Passarella[4], Lidia Bakota[2], Giulio Rastelli[1, *], Roland Brandt[2, 5, 6, *]

[1]Department of Life Sciences, University of Modena and Reggio Emilia, Italy.
[2]Department of Neurobiology, School of Biology/Chemistry, Osnabrück University, Germany.
[3]Department of Neurobiology, Interdisciplinary Center for Neurosciences, Heidelberg University, Germany.
[4]Department of Chemistry, University of Milan, Italy.
[5]Center for Cellular Nanoanalytics, Osnabrück University, Germany.
[6]Institute of Cognitive Science, Osnabrück University, Germany.

[#]Both authors contributed equally to the work
*Co-corresponding authors:
giulio.rastelli@unimore.it; Phone: +39-059-205-8564
roland.brandt@uni-osnabrueck.de; Phone: +49-5441-969-2338

[λ]Present address: Drug Discovery Unit, Wellcome Centre for Anti-Infectives Research, Division of Biological Chemistry and Drug Discovery, College of Life Sciences, University of Dundee, Dundee, DD1 5EH, UK.
[Ω]Present address: Department of Food, Environmental and Nutritional Sciences (DeFENS), University of Milan, Italy.

Table of contents:

Ligand-based analyses

Structure-based analyses including TABLES A-C and FIGURES A-D

TABLES S1-S12

FIGURES S1-S22

REFERENCES

Ligand-based analyses

Preparation of the compounds: The ChEMBL database (release 23; accessed on: April 3rd, 2018) [1] was downloaded and filtered to retain only compounds with activity annotations expressed as IC_{50}, K_i, K_d, EC_{50} and Potency, and an assay confidence score higher than 8. Activity records were processed to remove potential false positive readouts and duplicates deriving by multiple experiments, retaining only those with the best value. Only records that derived by binding experiments on human proteins were retained, obtaining a total of 557188 unique ligands with activity towards 2208 targets. Afterwards, the PHOX and ChEMBL compounds were prepared as follows. The ionization and tautomeric states that are more likely to be observed for the ligands at pH equal to 7±2 were generated with *LigPrep* (Schrödinger) by using default settings (Schrödinger Release 2022-1: LigPrep; Schrödinger, LLC: New York, NY, USA, 2022). The structure of the compounds was also energetically minimized at this stage. Moreover, the ChEMBL ligands were also subjected to conformational sampling, operated with the default parameters of OMEGA2 software [2], generating up to 200 conformers *per* compound.

Similarity estimations and selection of the targets: A series of 2D and 3D similarity analyses were performed on the generated dataset of ChEMBL ligands by means of the MACCS and Circular (ECFP4) types of fingerprints (OpenEye Toolkits 2020.2.2 OpenEye Scientific Software, Santa Fe, NM. Available online: http://www.eyesopen.com), and ROCS (OpenEye) [3], respectively. The PHOX compounds were used as references in the 2D and 3D similarity calculations, which were conducted with the same settings we applied in previous studies [4]. The results of the 2D analyses were filtered to retain only similarities higher than 0.3 (ECFP4*fp*) and 0.8 (MACCS*fp*) in terms of Tanimoto, and *TanimotoCombo* score higher than 1.5, [5], obtaining a total of 813 and 183 records, respectively. Activity data related to the ChEMBL compounds that emerged from the analyses, was filtered to retain only records related to molecules acting through the same mechanism of action on the same target, and targets with at least three active ligands (*i.e.*, IC_{50}, K_i, K_d, EC_{50} or Potency < 1 µM) similar to PHOXs. Data analysis was performed by means of an *in-house* developed script implemented in the Pandas python library (https://conference.scipy.org/proceedings/scipy2010/pdfs/mckinney.pdf).

Analysis of the molecular descriptors: 118 different molecular descriptors implemented in the RDKit node of KNIME (https://dl.acm.org/doi/10.1145/1656274.1656280) were firstly calculated

for the ligands of the PHOX series, then compared with the most relevant properties extracted from the tau active ligands we have recently reported [4]. Moreover, we also prioritized the PHOX compounds according to the sum of squared distances (SSQD) of their properties, with respect to the median values of the most relevant descriptors of tau ligands (**Figure S2**). Besides, 10 selected molecular properties (*i.e.*, "*Rule Of Five*", "*Rule Of Three*", "*#rotor*", "*MW*", "*dipole*", "*QPPCaco*", "*QPlogBB*", "*QPPMDCK*", "*QPlogKhsa*" and "*PSA*") that help to describe drug-likeness, and blood-brain-barrier (BBB) permeability were also calculated with the *QikProp* (Schrödinger) software (Schrödinger Release 2022-1: QikProp; Schrödinger, LLC: New York, NY, USA, 2022) and compared with the property ranges of already approved drugs.

Structure-based analyses

System setup and molecular dynamics simulations of tau in the 4R-3R and 4R conformation: The Cryo-EM structure of the paired helical filament (PHF) fibril (PDB ID: 5O3L) [6], was used as initial structure of tau in 4R-3R conformation. The fibril is composed of two protofilaments (herein referred as protofilament A and protofilament B) each of which contains five stacked tau monomers, placed in a C-shaped manner (**Figure 7A**). The elucidated sequence of the tau monomer spans residues 306-378 (residues numbering refers to that of the 5O3L structure) organized in eight β-sheets interspersed by random coil regions, which constitute the so-called "core" of the PHF fold. Missing hydrogen atoms were added to the complexes using the internal coordinates of the Amber all-atom data base; all lysine and arginine residues were positively charged, while glutamine and aspartate residues negatively charged. Capping residues ACE and NMA were added to N-terminal Val306 and C-terminal Phe378, respectively, of each tau monomer in the PHF pentamer. All calculations in this study were performed with the Amber20 suite of programs (https://ambermd.org/doc12/Amber20.pdf), and the ff14SB force field [7] for the protein. The system was solvated in an octahedral box of TIP3P (https://pubs.acs.org/doi/abs/10.1021/j100384a009) water molecules centered on the ligand and extending 10 Å outside the protein on all sides. The CUDA version of PMEMD was used to perform all molecular mechanics and molecular dynamics (MD) calculations. The simulations employed a residue-based cut-off of 10 Å, a time step of 2 fs, and a constraint of bond lengths involving hydrogen atoms using the SHAKE [8] algorithm. The solvated complex was minimized

with 10000 steps of conjugate gradient minimization and equilibrated with MD at 300 K as follows: first, 100 ps at constant volume (NVP) with 2 kcal mol^{-1} $Å^{-2}$ restraint on the protein were performed in order to gradually heat the system from 0 K to 300 K without undesirable drifts of the structure. Then, a second equilibration step under constant pressure conditions (NPT at 1 atm) was performed for 100 ps, with 1 kcal $mol^{-1}Å^{-2}$ restraint on the same atoms, followed by additional 100 + 100 ps MD with gradually reduced restraints (0.5 and 0.2 kcal mol^{-1} $Å^{-2}$). Afterwards, the complexes were equilibrated for 40 ns, without restraints. After equilibration, a production run of 620 ns was performed on the PHF fibril, and coordinates were collected every 10 ps, resulting in a total of 62000 snapshots.

The simulations on the protofilament A alone and in complex with PHOX15 were performed using the same procedure detailed above, with a production run of 820 ns (82000 snapshots) and 800 ns (80000 snapshots), respectively.

The molecular mechanics (MM) parameters for PHOX15 (i.e., atom types and atomic charges) for the PHOX15 ligand were assigned with the Antechamber module of Amber20 (https://ambermd.org/doc12/Amber20.pdf). In particular, the ligands were assigned generalized amber force field (GAFF2) atom types and AM1-BCC atomic charges [9,10]. Missing force-field parameters were assigned with the PARMCHECK utility (https://ambermd.org/doc12/Amber20.pdf).

As for tau in the 4R conformation, the cryo-EM structure of tau in progressive supranuclear palsy (PDB ID 7P65 PDB) [11] was used as an additional starting conformation. The fibril includes one protofilaments containing five stacked tau monomers (**Figure 7D**). The elucidated sequence of the tau monomer spans residues 272-381 organized in twelve β-sheets interspersed by random coil regions. System setup was performed with the same settings described above, except for the capping residues ACE and NMA, which were added to N-terminal Gly272 and C-terminal Asn381, respectively, of each tau monomer in the protofilament. The MD simulations (both the equilibration and production phases) on the protofilament alone and in complex with PHOX15 were performed using the same procedure detailed above, collecting a total of 80000 snapshots (800 ns) for each production run. The parameters of the PHOX15 ligand were generated as detailed above. Neither folding aberrancies, nor convergency issues were observed during the simulations,

as evidenced from radius of gyration from RMSD plots evaluated over the simulation time (**Figures S18**).

Post-processing and analyses of the MD trajectories: All post-processing and analyses of the MD trajectories reported within this study were performed with the standard utilities available in the Amber20 and GROMACS 2021.5 suites of programs (double precision) [12], by using default settings. Conversion from AMBER-type trajectories (.nc) to the GROMACS-type trajectories (.xtc) format was performed with the MDconvert and ACPYPE python-based tools [13-15]. Moreover, analyses of the water molecules crossing the identified cryptic channels, and hydrogen bonds of protofilament A and of the protein-ligand system were evaluated by means of *in house* developed script implemented with the *prody* python library [16]. The coordinates of the residues lining the cryptic channels were used as references for the calculations of the number of waters crossing the pockets during the MD simulation. Statistical analyses of the Φ and ψ angles were performed with a combination of *in-house* bash and python scripts. Visual inspection and graphic representation of relevant snapshots were performed using a combination of VMD, Pymol, Chimera and Maestro [17,18] (Schrödinger Release 2021-1: Maestro, Schrödinger, LLC, New York, NY, 2021).

Identification and characterization of the cryptic pockets: In order to assess the presence of potentially druggable binding pockets in the structures sampled by the MD simulations, we used the MDpocket program [19] from the fpocket 4.0 suite of programs [20]. We performed a MDpocket analysis (exploration phase) after structural superposition and RMS fit (considering backbone carbon atoms) of the structural ensembles. Three density maps that span the whole tau pentamer (*e.g.*, with a channel-like shape) were found on protofilament A and four on protomer B (**Figure A**, *panel 1)* and *2)*) at isovalue in the range of 0.08-0.3. Afterwards, we have run the MDpocket scoring calculation (characterization phase) on the selected pockets to evaluate their volume and surface accessible solvent area (SASA) evolution during the simulation time.

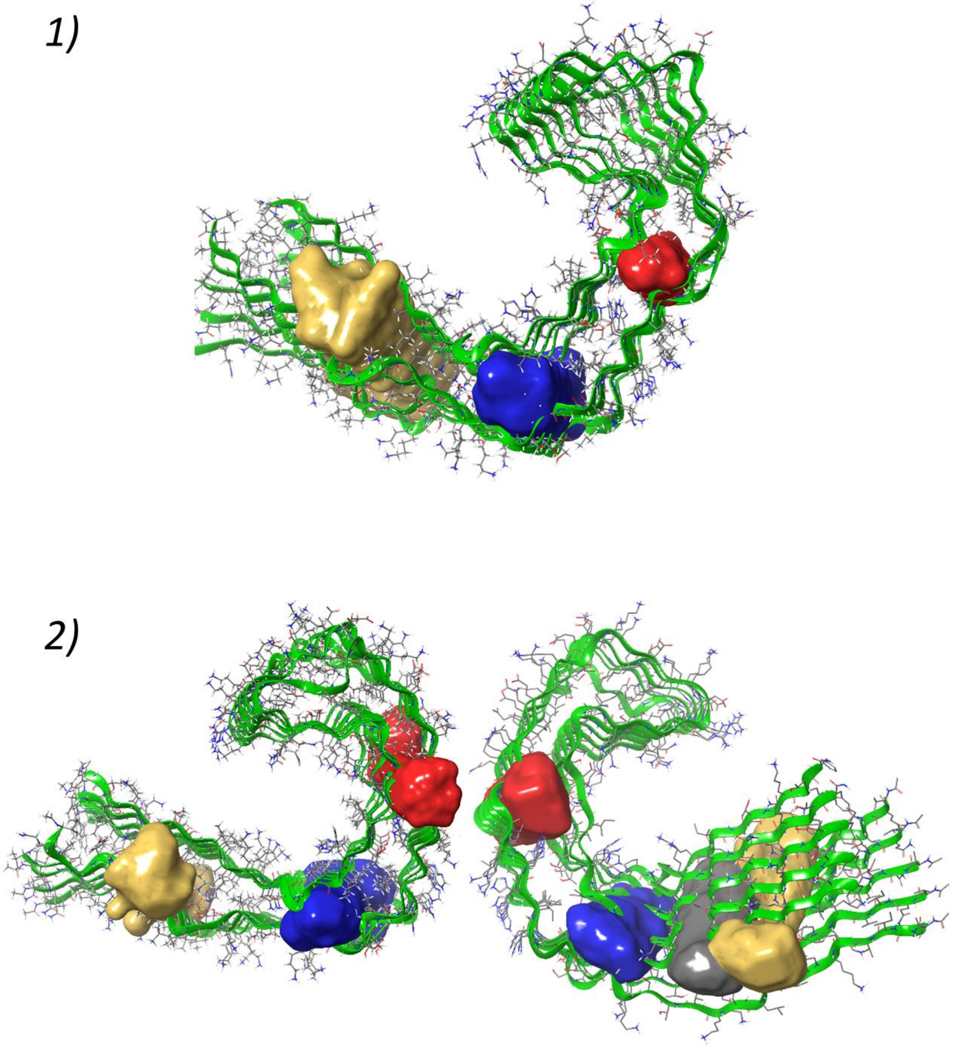

Figure A: Density maps of the cryptic pockets identified in the protofilament A and on the PHF dimer (PDB structure: 5O3L) using MDpocket. ***Panel 1***): Pocket 2 is shown in red, pocket 3 in blue and pocket 1 in yellow. Pocket densities span the whole fibril pentamer. ***Panel 2***): Pocket 2 and 2* are shown in red, pocket 4* in grey, pocket 3 and 3* in blue and pocket 1 and 1* in yellow. Pocket densities span the whole fibril pentamer.

A careful structural superposition of the MD ensembles considering all backbone atoms was performed prior to the pocket analyses with MDpocket/fpocket. According to literature data, structural superposition considering only the residues that shape a given pocket of interest could be used to improve scoring accuracy [19]. However, calculations performed using this latter approach resulted in severe errors during the analysis related to high fluctuations of the superposed snapshots. This is likely due to the disordered and flexible nature of the PHF structure, compared to “classic” globular proteins or enzymes commonly used in MDpocket validation papers. Both

exploration and pocket characterization analyses were carried out using parameters specific for ion channels and small cavities, with the maximum alpha sphere size, the minimum alpha sphere size and number of alpha spheres set to values of 5.5, 2.8 and 3, respectively. Statistical analysis, whose results are reported in **Table S7** (see below), was performed using *in-house* bash scripts.

Druggability analysis: Estimation of the druggable properties of the previously selected pockets was performed with MDpocket; exploration runs were performed by filtering the results according to the druggability score formalism proposed in fpocket [21]. Accordingly, to determine the druggable propensity of our pockets we have used the standard parameters (**Figure S15**); for pocket 2 and pocket 2* we observed a similar trend with that of runs performed with channel-type parameters, a situation that makes our data consistent with the speculation that these two pockets display the higher druggable likeliness.

Analysis of pockets' shape: The radius of the most interesting pockets identified by the previous MDpocket analysis was calculated by means of the HOLE [22] program. Due to the mainly disordered (random coil) nature of the PHF pentamer, we provided as input – for each snapshot – only residues forming the pocket of interest rather than the whole structure. Each snapshot was aligned with respect to the y axis. As such, we allowed the program to guess the center of the channel for its MonteCarlo grid search, so that to avoid any bias due to the flexible nature of the PHF structure. The MonteCarlo number of steps for each HOLE run was set to 30000; the step length and the Boltzmann factor were set to 0.1 Å and 0.2 Å, respectively, and the cutoff for the MonteCarlo search was set to 5.0 Å. We have used 1/10 of the total snapshots obtained from the MD simulations; for each snapshot we performed six HOLE replicates with different random seeds (generated randomly from 0 to 32767), so that to have a reliable and consistent statistics for the estimation of the pocket shape and the averaged pore radius. Averaging, smoothing and standard deviation estimations were performed using *in-house* bash scripts.

Note on 5O3L (4R-3R tau) residues indexing: The 5O3L Cryo-EM structure of tau PHF features tau residues 306-378 for each monomeric protein. Accordingly, each of the five stacked monomers spans the same index range (306-378), which makes structural and atomistic characterization of MD ensembles quite a challenging task.

Therefore, in order to overcome this limitation and to isolate each specific residue contribution in a given analysis, we re-indexed all residues as follows: the ACE residue of the first monomer of

protofilament A was indexed as 1, whereas the corresponding NMA residue of the same monomer was indexed as 75. Then, the ACE and NMA residues of the first monomer of protofilament B (i.e., the one interacting with the previous monomer of protofilament A) were indexed as 76 and 150, respectively. The same procedure applies then to the second monomer of protofilament A and so on for the rest of the structure, with a final residue index range of 1-750. Accordingly, all Tables and analyses presented in the following sections follow the residue indexing scheme illustrated above. Residue indexes for the pockets characterized in this work are shown in **Table A**.

Table A: Residue indexes for each identified pocket within the PHF dimer.

Residue	***Pocket***	***MD residue indexing***	***5O3L residue indexing***	***Filament***	***Element***
Pro	P1	8	312	1	channel
Val	P1	9	313	1	channel
Asp	P1	10	314	1	channel
Ser	P4	16	320	1	channel
Lys	P4	17	321	1	channel
Cys	P4	18	322	1	channel
Gly	P4	19	323	1	channel
Ser	P4	20	324	1	channel
Leu	P4	21	325	1	channel
**Pro**	**P2**	**28**	**332**	**1**	**PGGGQ***
**Gly**	**P2**	**29**	**333**	**1**	**PGGGQ***
**Gly**	**P2**	**30**	**334**	**1**	**PGGGQ***
**Gly**	**P2**	**31**	**335**	**1**	**PGGGQ***
Leu	P2	53	357	1	channel
Asp	P2	54	358	1	channel
Asn	P2	55	359	1	channel
Val	P4	59	363	1	channel
Pro	P4	60	364	1	channel
Gly	P4	61	365	1	channel
Gly	P4	62	366	1	channel
Lys	P1	66	370	1	channel

Glu	P1	68	372	1	channel
His	P1	70	374	1	channel
Pro	P1*	83	312	1	channel
Val	P1*	84	313	1	channel
Asp	P1*	85	314	1	channel
Asp	P3*	85	314	1	channel
Ser	P3*	87	316	1	channel
Lys	P3*	88	317	1	channel
Val	P3*	89	318	1	channel
Ser	P4*	91	320	1	channel
Lys	P4*	92	321	1	channel
Cys	P4*	93	322	1	channel
Gly	P4*	94	323	1	channel
Ser	P4*	95	324	1	channel
Leu	P4*	96	325	1	channel
**Pro**	**P2***	**103**	**332**	**1**	**PGGGQ***
**Gly**	**P2***	**104**	**333**	**1**	**PGGGQ***
**Gly**	**P2***	**105**	**334**	**1**	**PGGGQ***
**Gly**	**P2***	**106**	**335**	**1**	**PGGGQ***
Leu	P2*	128	357	1	channel
Asp	P2*	129	358	1	channel
Asn	P2*	130	359	1	channel
Val	P4*	134	363	1	channel
Pro	P4*	135	364	1	channel
Gly	P4*	136	365	1	channel
Gly	P4*	137	366	1	channel
Asn	P3*	139	368	1	channel
Lys	P3*	140	369	1	channel
Lys	P1*	141	370	1	channel
Lys	P3*	141	370	1	channel
Glu	P1*	143	372	1	channel
His	P1*	145	374	1	channel

Pro	P1	158	312	2	channel
Val	P1	159	313	2	channel
Asp	P1	160	314	2	channel
Ser	P4	166	320	2	channel
Lys	P4	167	321	2	channel
Cys	P4	168	322	2	channel
Gly	P4	169	323	2	channel
Ser	P4	170	324	2	channel
Leu	P4	171	325	2	channel
**Pro**	**P2**	**178**	**332**	**2**	**PGGGQ***
**Gly**	**P2**	**179**	**333**	**2**	**PGGGQ***
**Gly**	**P2**	**180**	**334**	**2**	**PGGGQ***
**Gly**	**P2**	**181**	**335**	**2**	**PGGGQ***
Leu	P2	203	357	2	channel
Asp	P2	204	358	2	channel
Asn	P2	205	359	2	channel
Val	P4	209	363	2	channel
Pro	P4	210	364	2	channel
Gly	P4	211	365	2	channel
Gly	P4	212	366	2	channel
Lys	P1	216	370	2	channel
Glu	P1	218	372	2	channel
His	P1	220	374	2	channel
Pro	P1*	233	312	2	channel
Val	P1*	234	313	2	channel
Asp	P1*	235	314	2	channel
Asp	P3*	235	314	2	channel
Ser	P3*	237	316	2	channel
Lys	P3*	238	317	2	channel
Val	P3*	239	318	2	channel
Ser	P4*	241	320	2	channel
Lys	P4*	242	321	2	channel

Cys	P4*	243	322	2	channel
Gly	P4*	244	323	2	channel
Ser	P4*	245	324	2	channel
Leu	P4*	246	325	2	channel
Pro	P2*	253	332	2	**PGGGQ***
Gly	P2*	254	333	2	**PGGGQ***
Gly	P2*	255	334	2	**PGGGQ***
Gly	P2*	256	335	2	**PGGGQ***
Leu	P2*	278	357	2	channel
Asp	P2*	279	358	2	channel
Asn	P2*	280	359	2	channel
Val	P4*	284	363	2	channel
Pro	P4*	285	364	2	channel
Gly	P4*	286	365	2	channel
Gly	P4*	287	366	2	channel
Asn	P3*	289	368	2	channel
Lys	P3*	290	369	2	channel
Lys	P1*	291	370	2	channel
Lys	P3*	291	370	2	channel
Glu	P1*	293	372	2	channel
His	P1*	295	374	2	channel
Pro	P1	308	312	3	channel
Val	P1	309	313	3	channel
Asp	P1	310	314	3	channel
Ser	P4	316	320	3	channel
Lys	P4	317	321	3	channel
Cys	P4	318	322	3	channel
Gly	P4	319	323	3	channel
Ser	P4	320	324	3	channel
Leu	P4	321	325	3	channel
Pro	P2	328	332	3	**PGGGQ***
Gly	P2	329	333	3	**PGGGQ***

Gly	P2	330	334	3	**PGGGQ***
Gly	P2	331	335	3	**PGGGQ***
Leu	P2	353	357	3	channel
Asp	P2	354	358	3	channel
Asn	P2	355	359	3	channel
Val	P4	359	363	3	channel
Pro	P4	360	364	3	channel
Gly	P4	361	365	3	channel
Gly	P4	362	366	3	channel
Lys	P1	366	370	3	channel
Glu	P1	368	372	3	channel
His	P1	370	374	3	channel
Pro	P1*	383	312	3	channel
Val	P1*	384	313	3	channel
Asp	P1*	385	314	3	channel
Asp	P3*	385	314	3	channel
Ser	P3*	387	316	3	channel
Lys	P3*	388	317	3	channel
Val	P3*	389	318	3	channel
Ser	P4*	391	320	3	channel
Lys	P4*	392	321	3	channel
Cys	P4*	393	322	3	channel
Gly	P4*	394	323	3	channel
Ser	P4*	395	324	3	channel
Leu	P4*	396	325	3	channel
**Pro**	**P2***	**403**	**332**	**3**	**PGGGQ***
**Gly**	**P2***	**404**	**333**	**3**	**PGGGQ***
**Gly**	**P2***	**405**	**334**	**3**	**PGGGQ***
**Gly**	**P2***	**406**	**335**	**3**	**PGGGQ***
Leu	P2*	428	357	3	channel
Asp	P2*	429	358	3	channel
Asn	P2*	430	359	3	channel

Val	P4*	434	363	3	channel
Pro	P4*	435	364	3	channel
Gly	P4*	436	365	3	channel
Gly	P4*	437	366	3	channel
Asn	P3*	439	368	3	channel
Lys	P3*	440	369	3	channel
Lys	P1*	441	370	3	channel
Lys	P3*	441	370	3	channel
Glu	P1*	443	372	3	channel
His	P1*	445	374	3	channel
Pro	P1	458	312	4	channel
Val	P1	459	313	4	channel
Asp	P1	460	314	4	channel
Ser	P4	466	320	4	channel
Lys	P4	467	321	4	channel
Cys	P4	468	322	4	channel
Gly	P4	469	323	4	channel
Ser	P4	470	324	4	channel
Leu	P4	471	325	4	channel
Pro	P2	478	332	4	channel
Gly	P2	479	333	4	channel
Gly	P2	480	334	4	channel
Gly	P2	481	335	4	channel
Leu	P2	503	357	4	channel
Asp	P2	504	358	4	channel
Asn	P2	505	359	4	channel
Val	P4	509	363	4	channel
Pro	P4	510	364	4	channel
Gly	P4	511	365	4	channel
Gly	P4	512	366	4	channel
Lys	P1	516	370	4	channel
Glu	P1	518	372	4	channel

His	P1	520	374	4	channel
Pro	P1*	533	312	4	channel
Val	P1*	534	313	4	channel
Asp	P1*	535	314	4	channel
Asp	P3*	535	314	4	channel
Ser	P3*	537	316	4	channel
Lys	P3*	538	317	4	channel
Val	P3*	539	318	4	channel
Ser	P4*	541	320	4	channel
Lys	P4*	542	321	4	channel
Cys	P4*	543	322	4	channel
Gly	P4*	544	323	4	channel
Ser	P4*	545	324	4	channel
Leu	P4*	546	325	4	channel
**Pro**	**P2***	**553**	**332**	**4**	**PGGGQ***
**Gly**	**P2***	**554**	**333**	**4**	**PGGGQ***
**Gly**	**P2***	**555**	**334**	**4**	**PGGGQ***
**Gly**	**P2***	**556**	**335**	**4**	**PGGGQ***
Leu	P2*	578	357	4	channel
Asp	P2*	579	358	4	channel
Asn	P2*	580	359	4	channel
Val	P4*	584	363	4	channel
Pro	P4*	585	364	4	channel
Gly	P4*	586	365	4	channel
Gly	P4*	587	366	4	channel
Asn	P3*	589	368	4	channel
Lys	P3*	590	369	4	channel
Lys	P1*	591	370	4	channel
Lys	P3*	591	370	4	channel
Glu	P1*	593	372	4	channel
His	P1*	595	374	4	channel
Pro	P1	608	312	5	channel

Val	P1	609	313	5	channel
Asp	P1	610	314	5	channel
Ser	P4	616	320	5	channel
Lys	P4	617	321	5	channel
Cys	P4	618	322	5	channel
Gly	P4	619	323	5	channel
Ser	P4	620	324	5	channel
Leu	P4	621	325	5	channel
Pro	P2	628	332	5	**PGGGQ***
Gly	P2	629	333	5	**PGGGQ***
Gly	P2	630	334	5	**PGGGQ***
Gly	P2	631	335	5	**PGGGQ***
Leu	P2	653	357	5	channel
Asp	P2	654	358	5	channel
Asn	P2	655	359	5	channel
Val	P4	659	363	5	channel
Pro	P4	660	364	5	channel
Gly	P4	661	365	5	channel
Gly	P4	662	366	5	channel
Lys	P1	666	370	5	channel
Glu	P1	668	372	5	channel
His	P1	670	374	5	channel
Pro	P1*	683	312	5	channel
Val	P1*	684	313	5	channel
Asp	P1*	685	314	5	channel
Asp	P3*	685	314	5	channel
Ser	P3*	687	316	5	channel
Lys	P3*	688	317	5	channel
Val	P3*	689	318	5	channel
Ser	P4*	691	320	5	channel
Lys	P4*	692	321	5	channel
Cys	P4*	693	322	5	channel

Gly	P4*	694	323	5	channel
Ser	P4*	695	324	5	channel
Leu	P4*	696	325	5	channel
Pro	P2*	703	332	5	**PGGGQ***
Gly	P2*	704	333	5	**PGGGQ***
Gly	P2*	705	334	5	**PGGGQ***
Gly	P2*	706	335	5	**PGGGQ***
Leu	P2*	728	357	5	channel
Asp	P2*	729	358	5	channel
Asn	P2*	730	359	5	channel
Val	P4*	734	363	5	channel
Pro	P4*	735	364	5	channel
Gly	P4*	736	365	5	channel
Gly	P4*	737	366	5	channel
Asn	P3*	739	368	5	channel
Lys	P3*	740	369	5	channel
Lys	P1*	741	370	5	channel
Lys	P3*	741	370	5	channel
Glu	P1*	743	372	5	channel
His	P1*	745	374	5	channel

Note on 7P65 (4R tau) residues indexing: The structure features tau residues 272-381 for each monomeric protein, making the structural and atomistic characterizations of MD ensembles challenging, as also observed for 5O3L. Therefore, we re-indexed all residues as previously performed for the 5O3L structure, the final residue index range resulting 1-565. **Table B** reports the residues lining pocket P5 shown in **Figure 5D**.

Table B: Correspondences between the 7P65 residues index of P5 and those from the MD simulations.

Residue	***MD residue indexing***	***7P65 residue indexing***	***Filament***	***Element***	***Residue***	***MD residue indexing***	***7P65 residue indexing***	***Filament***	***Element***
Ser	272	316	1	channel	Ser	385	316	4	channel
Lys	273	317	1	channel	Lys	386	317	4	channel
Val	274	318	1	channel	Val	387	318	4	channel

Thr	275	319	1	channel	Thr	388	319	4	channel
Ser	276	320	1	channel	Ser	389	320	4	channel
Lys	277	321	1	channel	Lys	390	321	4	channel
Cys	278	322	1	channel	Cys	391	322	4	channel
Gly	279	323	1	channel	Gly	392	323	4	channel
Ser	280	324	1	channel	Ser	393	324	4	channel
Lys	281	325	1	channel	Lys	394	325	4	channel
Gly	282	326	1	channel	Gly	395	326	4	channel
Asn	283	327	1	channel	Asn	396	327	4	channel
Pro	288	332	**1**	PGGGQ*	Pro	401	332	**4**	PGGGQ*
Gly	289	333	**1**	PGGGQ*	Gly	402	333	**4**	PGGGQ*
Gly	290	334	**1**	PGGGQ*	Gly	403	334	**4**	PGGGQ*
Gly	291	335	**1**	PGGGQ*	Gly	404	335	**4**	PGGGQ*
Gln	292	336	**1**	PGGGQ*	Gln	405	336	**4**	PGGGQ*
Glu	294	338	1	channel	Glu	407	338	4	channel
Val	295	339	1	channel	Val	408	339	4	channel
Lys	296	340	1	channel	Lys	409	340	4	channel
Ser	297	341	1	channel	Ser	410	341	4	channel
Glu	298	342	1	channel	Glu	411	342	4	channel
Lys	299	343	1	channel	Lys	412	343	4	channel
Ser	159	316	2	channel	Ser	498	316	5	channel
Lys	160	317	2	channel	Lys	499	317	5	channel
Val	161	318	2	channel	Val	500	318	5	channel
Thr	162	319	2	channel	Thr	501	319	5	channel
Ser	163	320	2	channel	Ser	502	320	5	channel
Lys	164	321	2	channel	Lys	503	321	5	channel
Cys	165	322	2	channel	Cys	504	322	5	channel
Gly	166	323	2	channel	Gly	505	323	5	channel
Ser	167	324	2	channel	Ser	506	324	5	channel

Lys	168	325	2	channel
Gly	169	326	2	channel
Asn	170	327	2	channel
Pro	175	332	**2**	**PGGGQ***
Gly	176	333	**2**	**PGGGQ***
Gly	177	334	**2**	**PGGGQ***
Gly	178	335	**2**	**PGGGQ***
Gln	179	336	**2**	**PGGGQ***
Glu	181	338	2	channel
Val	182	339	2	channel
Lys	183	340	2	channel
Ser	184	341	2	channel
Glu	185	342	2	channel
Lys	186	343	2	channel
Ser	46	316	3	channel
Lys	47	317	3	channel
Val	48	318	3	channel
Thr	49	319	3	channel
Ser	50	320	3	channel
Lys	51	321	3	channel
Cys	52	322	3	channel
Gly	53	323	3	channel
Ser	54	324	3	channel
Lys	55	325	3	channel
Gly	56	326	3	channel
Asn	57	327	3	channel
Pro	62	332	**3**	**PGGGQ***
Gly	63	333	**3**	**PGGGQ***
Lys	507	325	5	channel
Gly	508	326	5	channel
Asn	509	327	5	channel
Pro	514	332	**5**	**PGGGQ***
Gly	515	333	**5**	**PGGGQ***
Gly	516	334	**5**	**PGGGQ***
Gly	517	335	**5**	**PGGGQ***
Gln	518	336	**5**	**PGGGQ***
Glu	520	338	5	channel
Val	521	339	5	channel
Lys	522	340	5	channel
Ser	523	341	5	channel
Glu	524	342	5	channel
Lys	525	343	5	channel

Gly	64	334	**3**	**PGGGQ ***
Gly	65	335	**3**	**PGGGQ ***
Gln	66	336	**3**	**PGGGQ ***
Glu	68	338	3	channel
Val	69	339	3	channel
Lys	70	340	3	channel
Ser	71	341	3	channel
Glu	72	342	3	channel
Lys	73	343	3	channel

Note: * PGGGQ refers to the glycine triads and the adjacent proline and glutamine residues that are likely to be involved in the formation of PHF.

Analysis of the *glycine triads*: An analysis of the tau protofilament A MD trajectory was also performed to evaluate the structural variability of the glycines lining the identified cryptic pockets. In particular, our attention was focused on pocket 2, which resulted the most promising among those identified in this study. To this aim, the Φ and ψ angles of the *glycine triads* lining pocket 2 (*i.e.*, residues 29-31, 104-106, 179-181, 254-256 and 329-331) were firstly calculated by using the *CCPTRAJ* module available in Amber20 (https://ambermd.org/doc12/Amber20.pdf). Then, their distribution over the MD simulation time was evaluated through analysis of the respective Ramachandran and heatmap plots (**Figure S14**). The plots were generated by means of a script implemented in R (R Core Team, 2021). The conformational stability of the glycine residues lining the pocket 2 was also assessed by visual inspection of the tau MD, and a trajectory statistical analysis of the Φ and ψ angles (**Table C**).

Table C: Statistical analysis of the *glycine triads* Φ and ψ angles. The glycine Φ and ψ angles are scaled in the 0-360° range.

	Residue / angle	Min angle	Max angle	Median angle	Mean angle	Std. deviation	Variance
Filament 1	29 Φ	19.05	335.23	178.99	187.63	99.12	9825.32
	29 ψ	0.00	359.99	181.45	186.31	53.53	2865.99
	30 Φ	21.47	336.22	190.44	185.29	90.55	8198.57
	30 ψ	0.00	359.99	176.99	185.54	67.00	4489.30
	31 Φ	18.04	332.27	87.49	128.18	76.35	5829.13
	31 ψ	0.01	359.98	183.55	184.76	27.16	737.76
Filament 2	104 Φ	165.66	337.95	289.00	288.42	11.53	133.00
	104 ψ	0.18	359.95	157.42	159.78	24.49	599.97
	105 Φ	36.03	342.02	284.73	278.80	29.88	893.10
	105 ψ	0.15	359.55	145.88	158.38	57.25	3277.18
	106 Φ	18.05	333.68	207.61	207.22	73.10	5343.62
	106 ψ	1.29	346.87	175.20	175.94	25.77	664.11
Filament 3	179 Φ	195.75	331.27	287.38	286.83	11.14	124.19
	179 ψ	0.03	359.99	160.70	168.63	47.89	2293.26
	180 Φ	26.56	356.99	285.06	267.79	57.58	3314.92
	180 ψ	0.04	359.98	139.03	149.47	42.24	1784.10
	181 Φ	21.79	333.58	251.02	227.83	58.14	3380.36
	181 ψ	33.51	305.73	174.23	174.46	23.88	570.08
Filament 4	254 Φ	161.38	337.69	282.67	281.99	12.11	146.76
	254 ψ	0.00	360.00	170.96	191.52	87.34	7628.35
	255 Φ	24.60	345.30	277.95	223.70	93.64	8769.03
	255 ψ	11.54	344.16	152.79	158.87	35.01	1225.39
	256 Φ	44.31	334.85	227.94	221.24	49.06	2407.12
	256 ψ	23.49	344.69	171.67	170.51	23.01	529.53
Filament 5	329 Φ	3.16	349.91	213.14	199.17	40.99	1680.45
	329 ψ	0.30	359.93	159.57	144.20	53.79	2893.67
	330 Φ	0.40	359.51	157.31	151.84	38.34	1470.20
	330 ψ	25.25	288.59	141.28	145.66	38.88	1512.02
	331 Φ	78.09	314.16	182.63	185.54	29.33	860.33
	331 ψ	59.59	304.95	167.68	170.98	37.92	1437.71

The percentage of occurrence of the Φ and ψ angles adopting a PHF-like conformation was also calculated for the *glycine triads* in both simulations of the PHF dimer (monomer A), protofilament A alone and protofilament A with bound PHOX15 as follows. For each tau filament, the Φ and ψ angles of the glycines triads were firstly calculated with the *CCPTRAJ* module, as described above. Then, the MD trajectory frames were filtered to retain only those with all the Φ and ψ angles of the three glycines ranging ± 30° around the values observed for the corresponding residues in the 5O3L cryo EM structure. The percentage of occurrence of the Φ and ψ angles was finally calculated for each tau filament by means of the following formula ((number of frames with the Φ

and ψ angles of the glycines in the filament adopting a PHF-like conformation / total number of frames of the simulation) ° 100). The occupancy (%) of the h-bond interactions was also evaluated for the same residues in each tau filament, following a similar approach (the angles and distances of the h-bond interactions were defined according to AMBER20 parameters). The root mean-square fluctuation (RMSF), and Φ and ψ angles variability plots of the glycine residues are reported in **Figure B**.

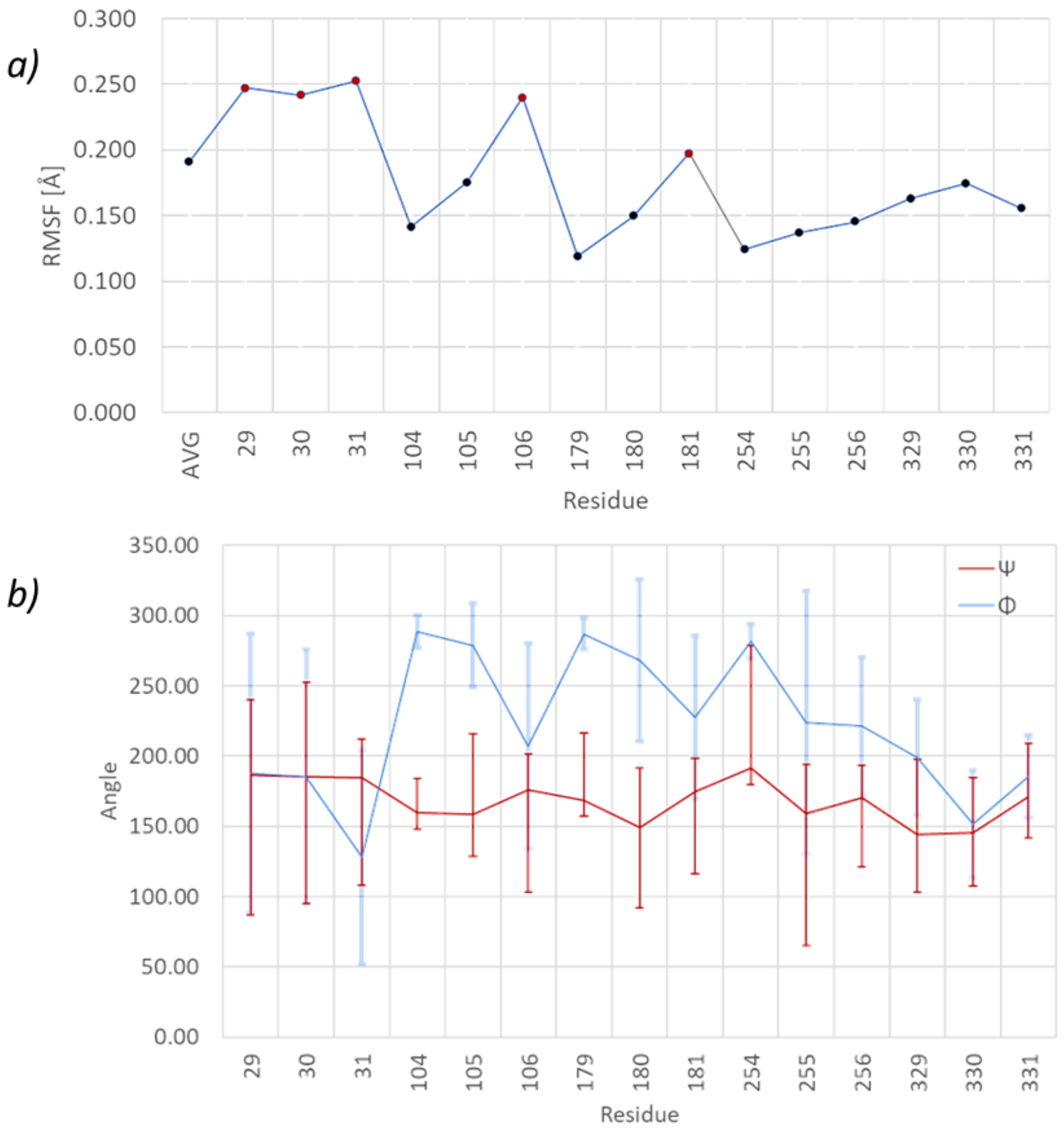

Figure B: RMSF fluctuations of the glycine residues lining the cryptic pocket 2 (panel *a)*) and averaged values of Φ and ψ angles observed for the same residues during the MD simulation (panel *b)*). In panel *a)*, RMSF values above the average of all the tau protofilament are highlighted in red. In panel *b)*, standard deviation of the glycine Φ and ψ angles, which are scaled in the 0-360° range,

are represented with bars. Residues numbering refers to that reported in **Table A**. The plots were generated with Excel of Microsoft (Microsoft Corporation 2018, Microsoft Excel. Retrieved from https://office.microsoft.com/excel).

Considering the high variability observed for the Φ and ψ angles of the *glycine triads* during the MD simulation, we performed an analysis of the druggability and pocket volume with MDpocket, according to modalities previously described.

Analysis of the glycine triads in the 7P65 PDB structure: Besides the analyses on 5O3L, the percentage of occurrence of the Φ and ψ angles adopting a PHF-like conformation was also calculated for the *glycine triads* in the protofilament alone and the protofilament bound to PHOX15 of the 7P65 cryo-EM structure, following the same procedure described above. Similarly, the same applies for the occupancy (%) of the h-bond interactions for the same residues in each tau filament. The RMSF calculated for the glycine triads and the residues lining the central channel-like pocket present in 7P65 are reported in **Figure C**.

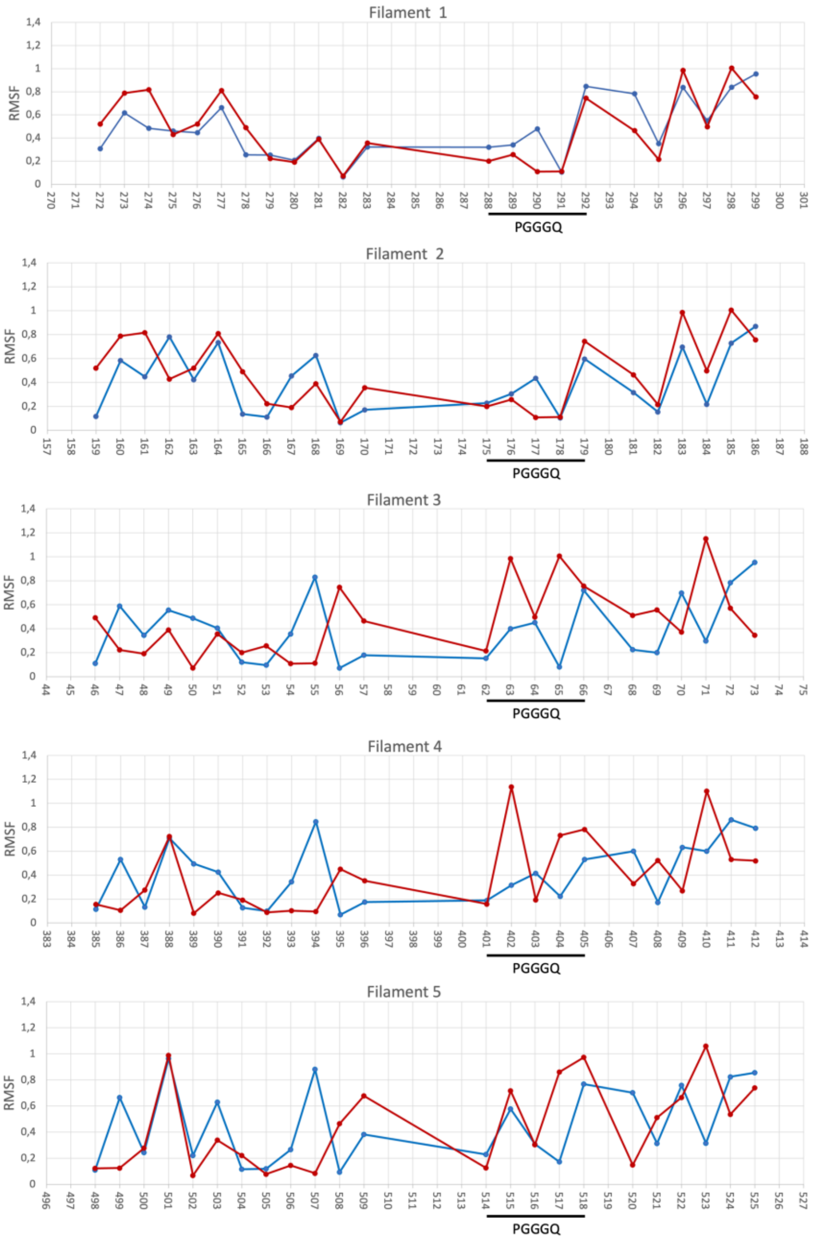

Figure C: RMSF fluctuations of glycine triads and the residues lining the central channel-like pocket of 7P65. RMSF fluctuations are reported for the residues from both the MD simulation of the protofilament alone (blue line) and the protofilament bound to PHOX15 (red line). Residues numbering refers to that reported in **Table B**. The plots were generated with Excel of Microsoft (Microsoft Corporation 2018, Microsoft Excel. Retrieved from https://office.microsoft.com/excel).

<u>Docking calculations of PHOX15 in tau structures</u>: Docking calculations were performed in selected representative structures sampled by MD by using the *Induced Fit Docking* protocol (*IFD*) implemented in Maestro (Schrödinger Release 2021-1: Induced Fit Docking protocol; Glide,

Schrödinger, LLC, New York, NY, 2021; Prime, Schrödinger, LLC, New York, NY, 2021). In the first stage of the protocol, receptor grids were generated with default settings and defined by 10 Å × 10 Å × 10 Å outer box placed at the centroids of pocket 2. While *IFD* calculations were conducted by using the default Glide SP mode with Van der Waals radii of the tau pocket 2 and of PHOX15 scaled by a factor of 0.8, as implemented in the default settings of the software. The subsequent step of refinement was performed on the top 30 structures within 100 kcal/mol of the best pose, and *Prime* was used to optimize the side chain of the residues lining pocket 2. Afterwards, the poses were redocked using the Glide XP mode and scored. The most relevant binding mode of PHOX15 was selected based on docking scores and visual inspection of the predicted poses.

Docking calculations in GSK3β: The docking of PHOX15 into GSK3β was performed with the *IDF* protocol with default settings, similarly to as previously described. In this case, the analyses were conducted into the ATP-binding site of the 4AFJ [23] crystal structure. The grid was generated with default parameters, by centering it on the coordinates of the co-crystallized compound SJJ. Prior to the docking calculations on PHOX15, the model was validated by redocking the co-crystallized ligand, obtaining a root mean-square deviation (RMSD) below 2.0 Å. The binding mode of PHOX15 into GSK3β was selected after visual inspection.

Docking calculations in Cdk5: The docking of PHOX15 into Cdk5 was performed with the *IDF* protocol with default settings, similarly to as previously described GSK3β. In this case, the analyses were conducted into the ATP-binding site of the 4AU8 crystal structure [24]. The grid was generated with default parameters, by centering it on the coordinates of the co-crystallized compound Z3R. In this case the conserved water W2089 was retained and considered as a part of the binding site during the docking calculations, this molecule participating also at the binding of the Z3R compound. Redocking the co-crystallized ligand was performed for validating the generated model. The binding mode of PHOX15 into Cdk5 was selected after visual inspection (**Figure D**).

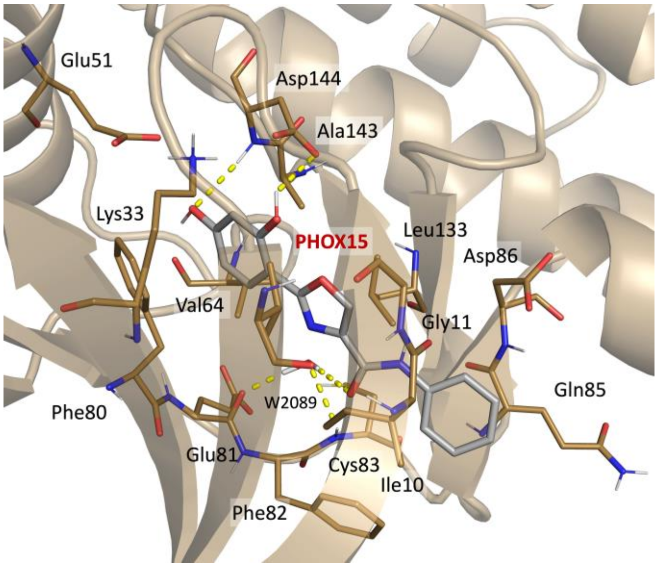

Figure D: Results of the docking calculations into Cdk5. The carbonyl of PHOX15 performs a H-bond interaction with a conserved water (i.e., W2089), which it also plays a key role for the binding of the co-crystallized ligand Z3R into Cdk5 (PDB ID: 4AU8). Moreover, the hydroxyl groups of resorcinol moiety establish H-bonds with the side chain and nitrogen backbone of Asp144. The phenyl ring of the same moiety is involved in π-π stacking interaction with the site chain of Phe180. While the phenyl moiety of PHOX15 was predicted to take place in proximity to the side chain of Ile10, Phe82 and Gln84 to establish hydrophobic interactions.

TABLES S1-S12

Table S1: Biological targets that resulted as promising according to the 2D and 3D ligand-based similarity analyses. Activity thresholds of 1 µM and 10 µM were used to discriminate active (IC_{50}, K_i, K_d, EC_{50} or Potency < 1 µM), moderately active (1 µM ≤ IC_{50}, K_i, K_d, EC_{50} or Potency ≤ 10 µM) and inactive (IC_{50}, K_i, K_d, EC_{50} or Potency > 10 µM) compounds reported in ChEMBL. The targets are ranked according to the number of PHOX compounds that showed similarity values above thresholds with their ChEMBL ligands. Targets emerging only from 2D fingerprints-based, 3D ROCS-based, or both similarity analyses are colored in black, blue, and red, respectively.

Target name	*# retrieved PHOXs*	*# Actives (< 1 µM)*	*# Moderately actives (1:10 µM)*	*# Inactives (> 10 µM)*
Inosine-5'-monophosphate dehydrogenase 2	12	40	11	0
Vascular endothelial growth factor receptor 2	12	17	7	1
Glycogen synthase kinase-3 beta	11	24	5	0
Microtubule-associated protein tau	***11***	***19***	***21***	***18***
Heat shock protein HSP 90-alpha	10	15	0	0
Pyruvate dehydrogenase kinase isoform 1	10	6	3	0
Serine/threonine-protein kinase Chk1	7	18	13	0
Diacylglycerol O-acyltransferase 1	7	14	1	0
Serine/threonine-protein kinase WEE1	6	28	3	0
Egl nine homolog 1	6	5	4	1
Cyclin-dependent kinase 2	6	4	5	5
Histone deacetylase 1	6	4	2	0
Peroxisome proliferator-activated receptor gamma	4	11	13	0
Peroxisome proliferator-activated receptor delta	4	8	4	0
Egl nine homolog 3	4	5	1	0
GABA receptor alpha-5 subunit	4	4	0	0
Metastin receptor	4	3	0	0
LDL-associated phospholipase A2	3	11	0	1
Thromboxane A2 receptor	3	8	0	0
Vascular endothelial growth factor receptor 1	3	3	0	0
Neuropeptide Y receptor type 5	2	17	0	0
Glucagon receptor	2	9	0	0

Table S2: For each PHOX compound, the tau ligands present in ChEMBL with similarity values above thresholds are reported. Only similarity records above thresholds (MACCS*fp* Tanimoto index ≥ 0.8, ECFP4*fp* Tanimoto index ≥ 0.3, 3D ROCS, *TanimotoCombo* score ≥ 1.5) with respect to tau ligands are reported. Several PHOX compounds showed significant similarity to a number of tau ChEMBL ligands. For example, CHEMBL1399653 (Potency = 398.1 nM) resulted similar to PHOX5, PHOX8, PHOX12, PHOX15, PHOX16, PHOX19 and PHOX20, while CHEMBL1319405 (Potency = 100 nM) was similar to PHOX5 and PHOX8. Moreover, one compound, *i.e.*, CHEMBL1514736 (Potency = 14125.4 nM), resulted similar to PHOX5 and PHOX8 according to 3D similarity analyses, and to PHOX15 based on 2D and 3D estimations.

**3D similarity estimations**					
Compound ID	***Tau ChEMBL ligand***	***Tanimoto Combo***	***Shape Tanimoto***	***Color Tanimoto***	***Potency (nM)***
PHOX5	CHEMBL1499585	1.604	0.907	0.697	5011.9
PHOX5	CHEMBL1514736	1.595	0.917	0.678	14125.4
PHOX5	CHEMBL1353188	1.586	0.884	0.702	3981.1
PHOX5	CHEMBL1601285	1.577	0.874	0.702	891.3
PHOX5	CHEMBL1315044	1.56	0.919	0.641	707.9
PHOX5	CHEMBL1483111	1.559	0.931	0.628	631
PHOX5	CHEMBL1513430	1.556	0.914	0.642	3162.3
PHOX5	CHEMBL1336847	1.552	0.923	0.629	631
PHOX5	CHEMBL1319405	1.549	0.919	0.63	100
PHOX5	CHEMBL1503784	1.54	0.917	0.623	14125.4
PHOX5	CHEMBL1302891	1.537	0.926	0.611	22387.2
PHOX5	CHEMBL1379136	1.537	0.908	0.629	707.9
PHOX5	CHEMBL1321832	1.532	0.902	0.63	5623.4
PHOX5	CHEMBL1569287	1.529	0.885	0.644	28183.8
PHOX5	CHEMBL1490856	1.526	0.884	0.642	14125.4
PHOX5	CHEMBL1570869	1.517	0.884	0.632	11220.2
PHOX5	CHEMBL1408001	1.51	0.884	0.625	1584.9
PHOX5	CHEMBL1404118	1.507	0.896	0.611	3162.3
PHOX5	CHEMBL1452108	1.507	0.896	0.611	14125.4
PHOX5	CHEMBL1458814	1.505	0.895	0.61	25118.9
PHOX5	CHEMBL1339445	1.502	0.865	0.637	3548.1
PHOX8	CHEMBL1499585	1.616	0.919	0.697	5011.9
PHOX8	CHEMBL1514736	1.61	0.932	0.678	14125.4
PHOX8	CHEMBL1353188	1.6	0.898	0.702	3981.1
PHOX8	CHEMBL1601285	1.59	0.888	0.702	891.3
PHOX8	CHEMBL1315044	1.588	0.947	0.641	707.9
PHOX8	CHEMBL1513430	1.578	0.936	0.642	3162.3
PHOX8	CHEMBL1321832	1.555	0.925	0.631	5623.4
PHOX8	CHEMBL1503784	1.554	0.936	0.618	14125.4
PHOX8	CHEMBL1569287	1.546	0.902	0.644	28183.8
PHOX8	CHEMBL1483111	1.542	0.914	0.628	631
PHOX8	CHEMBL1336847	1.536	0.907	0.628	631

PHOX8	CHEMBL1518128	1.532	0.954	0.578	316.2
PHOX8	CHEMBL1404118	1.532	0.922	0.61	3162.3
PHOX8	CHEMBL1570869	1.53	0.898	0.632	11220.2
PHOX8	CHEMBL1319405	1.529	0.902	0.628	100
PHOX8	CHEMBL1452108	1.525	0.915	0.61	14125.4
PHOX8	CHEMBL1408001	1.525	0.894	0.631	1584.9
PHOX8	CHEMBL1339445	1.525	0.888	0.637	3548.1
PHOX8	CHEMBL1458814	1.523	0.913	0.61	25118.9
PHOX8	CHEMBL1302891	1.521	0.911	0.61	22387.2
PHOX8	CHEMBL1379136	1.519	0.89	0.629	707.9
PHOX8	CHEMBL1427394	1.519	0.866	0.654	15848.9
PHOX8	CHEMBL1490856	1.517	0.876	0.642	14125.4
PHOX8	CHEMBL1458814	1.515	0.91	0.605	25118.9
PHOX8	CHEMBL1483649	1.51	0.863	0.647	2818.4
PHOX8	CHEMBL1516425	1.505	0.895	0.61	3162.3
PHOX15	CHEMBL1514736	1.505	0.919	0.586	14.125
PHOX15	CHEMBL1499585	1.501	0.901	0.600	5011.9
PHOX20	CHEMBL1514736	1.502	0.903	0.599	14.125
**2D similarity estimations**					
Compound ID	***Tau ChEMBL ligand***	**MACCS*fp* similarity score**	**ECFP4*fp*** ***similarity score***		***Potency (nM)***
PHOX5	CHEMBL1434761	0.896	0.793		1584.9
PHOX5	CHEMBL1465221	0.837	0.778		707.9
PHOX5	CHEMBL1484911	0.936	0.741		14125.4
PHOX5	CHEMBL1451527	0.812	0.704		223.9
PHOX5	CHEMBL1363420	0.812	0.655		15848.9
PHOX5	CHEMBL1591414	0.808	0.633		31622.8
PHOX5	CHEMBL1436351	0.878	0.594		354.8
PHOX5	CHEMBL1333222	0.804	0.531		1000
PHOX5	CHEMBL1399653	0.8	0.531		398.1
PHOX5	CHEMBL1437424	0.8	0.531		562.3
PHOX5	CHEMBL1578151	0.812	0.517		5623.4
PHOX5	CHEMBL1315438	0.854	0.516		501.2
PHOX5	CHEMBL1350958	0.812	0.515		31622.8
PHOX5	CHEMBL1488682	0.837	0.471		631
PHOX5	CHEMBL1374264	0.8	0.471		1258.9
PHOX5	CHEMBL1355303	0.837	0.457		707.9
PHOX5	CHEMBL1592402	0.837	0.457		794.3
PHOX5	CHEMBL1593426	0.8	0.457		398.1
PHOX5	CHEMBL1324008	0.833	0.429		794.3
PHOX5	CHEMBL1364841	0.812	0.412		3162.3
PHOX5	CHEMBL1457270	0.8	0.405		1258.9
PHOX5	CHEMBL1334889	0.804	0.4		31622.8
PHOX5	CHEMBL3189964	0.816	0.382		4466.8

PHOX5	CHEMBL1468693	0.812	0.343	25118.9
PHOX8	CHEMBL1545040	0.83	0.8	28183.8
PHOX8	CHEMBL1511045	0.812	0.8	1584.9
PHOX8	CHEMBL1434761	0.833	0.759	1584.9
PHOX8	CHEMBL1465221	0.891	0.741	707.9
PHOX8	CHEMBL1484911	0.872	0.704	14125.4
PHOX8	CHEMBL1333222	0.854	0.6	1000
PHOX8	CHEMBL1399653	0.851	0.6	398.1
PHOX8	CHEMBL1436351	0.816	0.515	354.8
PHOX8	CHEMBL1392298	0.8	0.5	22387.2
PHOX8	CHEMBL1329781	0.8	0.486	2818.4
PHOX8	CHEMBL1525488	0.809	0.457	3162.3
PHOX8	CHEMBL1603424	0.844	0.441	1122
PHOX8	CHEMBL3189964	0.87	0.394	4466.8
PHOX9	CHEMBL1434761	0.837	0.543	1584.9
PHOX9	CHEMBL1484911	0.875	0.531	14125.4
PHOX9	CHEMBL1465221	0.816	0.515	707.9
PHOX9	CHEMBL1436351	0.857	0.395	354.8
PHOX9	CHEMBL1315438	0.833	0.324	501.2
PHOX9	CHEMBL1488682	0.816	0.3	631
PHOX12	CHEMBL3198540	0.8	0.5	1000
PHOX12	CHEMBL1465221	0.848	0.485	707.9
PHOX12	CHEMBL1333222	0.812	0.471	1000
PHOX12	CHEMBL1399653	0.809	0.389	398.1
PHOX13	CHEMBL1417596	0.825	0.528	112.2
PHOX15	CHEMBL1514736	0.8	0.846	14125.4
PHOX15	CHEMBL1465221	0.854	0.815	707.9
PHOX15	CHEMBL1434761	0.837	0.767	1584.9
PHOX15	CHEMBL1511045	0.816	0.741	1584.9
PHOX15	CHEMBL1484911	0.837	0.714	14125.4
PHOX15	CHEMBL1399653	0.894	0.613	398.1
PHOX15	CHEMBL1436351	0.82	0.576	354.8
PHOX15	CHEMBL1445621	0.826	0.571	14125.4
PHOX15	CHEMBL1333222	0.82	0.562	1000
PHOX15	CHEMBL1329781	0.804	0.459	2818.4
PHOX15	CHEMBL1603424	0.809	0.417	1122
PHOX15	CHEMBL3189964	0.833	0.412	4466.8
PHOX16	CHEMBL1465221	0.812	0.545	707.9
PHOX16	CHEMBL3198540	0.804	0.514	1000
PHOX16	CHEMBL1399653	0.851	0.405	398.1
PHOX17	CHEMBL1417596	0.842	0.583	112.2
PHOX19	CHEMBL1484911	0.84	0.515	14125.4
PHOX19	CHEMBL1434761	0.84	0.486	1584.9
PHOX19	CHEMBL1436351	0.86	0.421	354.8

PHOX19	CHEMBL1315438	0.837	0.389	501.2
PHOX19	CHEMBL1399653	0.82	0.368	398.1
PHOX20	CHEMBL1465221	0.804	0.815	707.9
PHOX20	CHEMBL1484911	0.898	0.714	14125.4
PHOX20	CHEMBL1434761	0.898	0.71	1584.9
PHOX20	CHEMBL1591414	0.811	0.667	31622.8
PHOX20	CHEMBL1436351	0.88	0.625	354.8
PHOX20	CHEMBL1315438	0.857	0.6	501.2
PHOX20	CHEMBL1399653	0.84	0.562	398.1
PHOX20	CHEMBL1437424	0.804	0.562	562.3
PHOX20	CHEMBL1578151	0.816	0.5	5623.4
PHOX20	CHEMBL1488682	0.804	0.5	631
PHOX20	CHEMBL1592402	0.804	0.486	794.3
PHOX20	CHEMBL1350958	0.816	0.457	31622.8
PHOX20	CHEMBL1324008	0.8	0.457	794.3
PHOX20	CHEMBL1355303	0.804	0.405	707.9
PHOX27	CHEMBL1417596	0.8	0.5	112.2

Note: Activity data was retrieved from the ChEMBL Document ID CHEMBL1201862.

Table S3: Molecular descriptors of the PHOX compounds calculated as described in [4]. The median, and the 10th and 90th percentiles of the molecular descriptors of known tau anti-aggregation ligands are also reported for comparison.

Molecular descriptor	PHOX 5	PHOX 8	PHOX 9	PHOX 11	PHOX 12	PHOX 13	PHOX 15	PHOX 16	PHOX 17	PHOX 19	PHOX 20	PHOX 27	Median value	Perc. 10th actives	Perc. 90th actives
**SlogP**	3.95	3.30	5.22	3.07	4.57	2.42	3.01	4.27	2.12	4.93	3.66	2.77	*3.80*	*2.14*	*5.35*
**Num Lipinski HBA**	5	5	5	7	5	7	6	6	8	6	6	8	*6*	*4*	*9*
**Num Rotatable Bonds**	3	3	5	5	5	5	3	5	5	5	3	5	*5*	*2*	*8*
**Num HBA**	4	4	4	5	4	5	5	5	6	5	5	6	*5*	*3*	*8*
**Num Amide Bonds**	1	1	1	2	1	2	1	1	2	1	1	2	*1*	*0*	*2*
**Num Hetero Atoms**	6	5	6	8	5	7	6	6	8	7	7	9	*7*	*5*	*10*
**Num Rings**	3	3	4	3	4	3	3	4	3	4	3	3	*4*	*2*	*5*
**Num Aromatic Rings**	3	3	4	3	4	3	3	4	3	4	3	3	*3*	*2*	*4*
**Num Aliphatic Rings**	0	0	0	0	0	0	0	0	0	0	0	0	*0*	*0*	*2*
**Num Aromatic Heterocycles**	1	1	1	1	1	1	1	1	1	1	1	1	*1*	*0*	*2*
**Num Aromatic Carbocycles**	2	2	3	2	3	2	2	3	2	3	2	2	*2*	*1*	*3*
**Chi0v**	12.04	10.98	16.01	14.16	14.95	13.10	11.35	15.32	13.47	16.37	12.41	14.53	*15.74*	*12.13*	*19.78*
**Chi3n**	2.96	2.90	4.47	3.39	4.41	3.34	2.98	4.49	3.42	4.57	3.06	3.50	*4.35*	*2.96*	*5.95*
**HallKierAlpha**	-2.79	-3.08	-3.57	-3.32	-3.86	-3.61	-3.28	-4.06	-3.81	-3.77	-2.99	-3.52	*-3.07*	*-3.98*	*-1.90*
**kappa2**	5.91	5.50	8.20	7.71	7.80	7.31	5.61	7.90	7.39	8.31	6.02	7.79	*7.82*	*5.57*	*10.36*
**slogp_VSA1**	5.32	5.32	5.32	10.63	5.32	10.63	5.32	5.32	10.63	5.32	5.32	10.63	*10.05*	*4.72*	*19.08*
**slogp_VSA6**	59.21	65.28	89.54	59.21	95.61	65.28	59.21	89.54	59.21	83.48	53.14	53.14	*53.69*	*28.79*	*77.49*
**slogp_VSA8**	11.45	11.45	11.45	11.45	11.45	11.45	11.45	11.45	11.45	11.45	11.45	11.45	*10.90*	*0.00*	*22.55*
**slogp_VSA11**	5.75	5.75	5.75	5.75	5.75	5.75	11.50	11.50	11.50	11.50	11.50	11.50	*5.75*	*0.00*	*11.50*
**smr_VSA1**	14.32	14.32	14.32	19.11	14.32	19.11	19.42	19.42	24.22	19.42	19.42	24.22	*14.05*	*4.79*	*23.80*
**smr_VSA3**	4.98	4.98	10.30	10.30	10.30	10.30	4.98	10.30	10.30	10.30	4.98	10.30	*9.88*	*0.00*	*19.64*
**smr_VSA9**	17.20	17.20	17.20	17.20	17.20	17.20	22.95	22.95	22.95	22.95	22.95	22.95	*5.88*	*0.00*	*22.63*

**peoe_VSA1**	14.84	14.84	14.84	20.16	14.84	20.16	19.95	19.95	25.26	19.95	19.95	25.26	*10.63*	*4.74*	*20.11*
**peoe_VSA3**	4.98	4.98	4.98	4.98	4.98	4.98	4.98	4.98	4.98	4.98	4.98	4.98	*4.98*	*0.00*	*12.72*
**peoe_VSA7**	30.33	24.27	29.33	30.33	23.26	24.27	24.27	23.26	24.27	17.19	18.20	18.20	*43.17*	*23.77*	*63.61*
**peoe_VSA11**	5.69	5.69	5.69	5.69	5.69	5.69	5.69	5.69	5.69	5.69	5.69	5.69	*5.54*	*0.00*	*16.46*
**peoe_VSA12**	5.89	5.89	5.89	11.80	5.89	11.80	5.89	5.89	11.80	5.89	5.89	11.80	*5.75*	*0.00*	*11.81*
**peoe_VSA13**	5.91	5.91	5.91	5.91	5.91	5.91	5.91	5.91	5.91	5.91	5.91	5.91	*5.91*	*0.00*	*11.59*
**MQN10**	2	2	2	3	2	3	3	3	4	3	3	4	*3*	*1*	*5*
**MQN13**	6	5	8	9	7	8	6	8	9	9	7	10	*8*	*5*	*13*
**MQN27**	1	1	1	3	1	3	1	1	3	1	1	3	*3*	*1*	*6*
**MQN28**	1	1	2	2	2	2	1	2	2	2	1	2	*1*	*0*	*2*
**MQN30**	11	12	16	11	17	12	11	16	11	15	10	10	*11*	*7*	*15*
**MQN31**	6	5	7	6	6	5	6	7	6	8	7	7	*7*	*5*	*10*
**MQN36**	2	2	3	2	3	2	2	3	2	3	2	2	*3*	*1*	*4*
**MQN41**	0	0	0	0	0	0	0	0	0	0	0	0	*2*	*0*	*4*

Table S4: Sum of square distances (SSQDs) evaluated for the molecular descriptors of the PHOX compounds ($Desc._{PHOXs}$), with respect to the median value of the corresponding properties of reported tau active ligands ($Desc._{tau\ median}$) [4]. SSDQs were calculated according to the following equation:

$$\textbf{SSQD} = \sum(Desc._{tau\ median} - Desc._{PHOXs})^2$$

Compound ID	***Molecular descriptors SSQD***
PHOX5	430.57
PHOX11	493.38
PHOX8	749.95
PHOX13	798.23
PHOX15	921.60
PHOX17	1093.92
PHOX20	1142.93
PHOX27	1329.79
PHOX9	1688.85
PHOX19	2056.93
PHOX16	2183.69
PHOX12	2380.82

Table S5: Selected molecular descriptors related to the BBB permeability and drug-like properties evaluated with *QikProp* (Schrödinger). The compounds showed molecular properties in agreement with ranges and recommended values based on 95% of approved drugs. In particular, the compounds present a number of rotatable bonds lower than 6 [25], a dipole moment between 0 and 12.5, and a MW below 500. Moreover, the majority of the ligands showed also favorable values of the QPlogBB (predicted brain/blood partition coefficient, recommended range: -3.0 ÷ 1.2) and QPPMDCK (predicted apparent MDCK cell permeability in nm/sec., recommended range: >500 great; < 25 poor) descriptors.

Title	***Rule Of Five***	***Rule Of Three***	***#rotor***	***MW***	***dipole***	***QPP Caco***	***QP logBB***	***QPP MDCK***	***QP logKhsa***	***PSA***
PHOX5	0	0	3	314.7	6.7	1162.2	-0.4	1434.0	0.0	75.2
PHOX8	0	0	3	280.3	7.7	1161.8	-0.5	581.8	-0.1	75.2
PHOX9	0	1	5	404.9	6.5	1586.8	-0.5	2007.9	0.6	74.3
PHOX11	0	0	5	371.8	6.8	430.7	-1.1	490.4	0.0	112.6
PHOX12	0	1	5	370.4	6.9	1537.6	-0.6	787.6	0.5	74.3
PHOX13	0	0	5	337.3	7.1	430.5	-1.2	198.9	-0.1	112.7
PHOX15	0	0	4	296.3	7.6	353.8	-1.1	160.9	-0.2	97.7
PHOX16	0	0	6	386.4	6.4	483.0	-1.2	225.3	0.4	96.8
PHOX17	0	0	6	353.3	7.0	131.1	-1.9	55.0	-0.2	135.1
PHOX19	0	1	6	420.9	7.3	552.9	-1.0	610.3	0.5	95.9
PHOX20	0	0	4	330.7	7.2	405.0	-0.9	435.9	-0.1	96.8
PHOX27	0	0	6	387.8	7.4	150.1	-1.7	149.1	-0.1	134.3

Table S6: Average values of the main pockets descriptors calculated with MDPocket by using channels-related parameters. Relative standard deviations (rSD) are also reported.

	Volume ($Å^3$)	***rSD (%)***	***SASA ($Å^2$)***	***rSD (%)***	***$SASA^{pol}$ ($Å^2$)***	***rSD (%)***	***$SASA^{apo}$ ($Å^2$)***	***rSD (%)***
**Protofilament A of 5O3L**								
**P1**	478.7 ± 156.2	32.6	359.5 ± 111.7	31.1	197.8 ± 64.6	32.7	161.6 ± 55.9	34.6
**P2**	314.2 ± 105.8	33.7	232.1 ± 71.3	30.7	114.0 ± 38.7	33.9	118.1 ± 42.7	36.2
**P3**	304.3 ± 130.5	42.9	220.8 ± 84.8	38.4	65.9 ± 33.0	50.1	155.0 ± 59.4	38.3
**PHF dimer**								
**P1**	357.7 ± 115.0	32.1	269.8 ± 79.2	29.4	139.7 ± 47.3	33.9	130.1 ± 41.9	32.2
**P1***	323.3 ± 111.6	34.5	257.1 ± 81.0	31.5	146.9 ± 48.5	33.0	110.1 ± 38.9	35.3
**P2**	480.6 ± 126.8	26.4	357.5 ± 84.0	23.5	201.1 ± 42.6	21.2	156.4 ± 51.8	33.1
**P2***	540.2 ± 142.3	26.3	326.5 ± 77.1	23.6	157.9 ± 33.5	21.2	168.6 ± 59.3	35.2
**P3**	504.6 ± 170.6	33.8	340.0 ± 106.0	31.2	99.3 ± 40.4	40.7	240.7 ± 72.3	30.0
**P3***	309.8 ± 141.5	45.7	215.8 ± 96.0	44.5	79.3 ± 35.9	45.3	136.5 ± 69.2	50.7
**P4***	567.9 ± 84.1	14.8	344.4 ± 54.7	15.9	91.6 ± 25.2	27.5	252.8 ± 36.1	14.3

Table S7: Average radii of pocket 2 and pocket 2* resulting from the MD simulations of the protofilament A and the PHF dimer. Relative standard deviations (rSD) are reported.

	**Pore radius (Å)**	**rSD (%)**
**single Protofilament**		
**P2**	2.09 ± 0.53	24.3
**PHF dimer**		
**P2**	2.23 ± 0.45	20.2
**P2***	2.69 ± 0.67	24.9

Table S8: Protofilament A – protofilament B H-bonds and salt bridges occupancies over the course of the MD simulation on the 5O3L PHF dimer. Only occupancies above 5% were considered. The superscript BB indicates backbone atoms whereas superscript SC indicates side chain atoms.

Donor	*Acceptor*	*Occupancy (%)*	*Type*
**Protofilament A – protofilament B**			
Q257-NH^{sc}	K327-O^{bb}	88.6	H-bond
G480-NH^{bb}	G555-O^{bb}	84.6	H-bond
Q257-NH^{sc}	P178-O^{bb}	83.7	H-bond
G180-NH^{bb}	G255-O^{bb}	82.8	H-bond
G330-NH^{bb}	G405-O^{bb}	82.5	H-bond
Q557-NH^{sc}	P478-O^{bb}	81.2	H-bond
G630-NH^{bb}	G705-O^{bb}	80.2	H-bond
G30-NH^{bb}	G105-O^{bb}	78.9	H-bond
G105-NH^{bb}	G180-O^{bb}	77.0	H-bond
G255-NH^{bb}	G330-O^{bb}	73.8	H-bond
Q557-NH^{sc}	K627-O^{bb}	72.6	H-bond
G405-NH^{bb}	G480-O^{bb}	61.4	H-bond
Q482-NH^{sc}	K552-O^{bb}	57.1	H-bond
G555-NH^{bb}	G630-O^{bb}	52.0	H-bond
Q707-NH^{sc}	P628-O^{bb}	49.7	H-bond
Q632-NH^{sc}	K702-O^{bb}	45.6	H-bond
Q332-NH^{sc}	K402-O^{bb}	42.8	H-bond
Q107-NH^{sc}	K177-O^{bb}	42.5	H-bond
Q107-NH^{sc}	P28-O^{bb}	37.0	H-bond
Q182-NH^{sc}	K252-O^{bb}	36.5	H-bond
Q407-NH^{sc}	P328-O^{bb}	30.9	H-bond
Q407-NH^{sc}	K477-O^{bb}	27.2	H-bond
Q32-NH^{sc}	K102-O^{bb}	23.3	H-bond
Q632-NH^{sc}	P553-O^{bb}	14.3	H-bond
G104-NH^{bb}	Q32-O^{sc}	11.7	H-bond
H625-NH^{sc}	E709-O^{sc}	8.8	H-bond
K702-NH^{sc}	Q632-O^{sc}	5.5	H-bond
K27-NH^{sc}	E109-O^{sc}	67.3	Salt bridge
K102-NH^{sc}	E34-O^{sc}	64.2	Salt bridge
K177-NH^{sc}	E259-O^{sc}	61.4	Salt bridge
K102-NH^{sc}	E184-O^{sc}	61.2	Salt bridge
K327-NH^{sc}	E409-O^{sc}	48.7	Salt bridge
K252-NH^{sc}	E334-O^{sc}	45.7	Salt bridge
K402-NH^{sc}	E484-O^{sc}	39.5	Salt bridge
K252-NH^{sc}	E184-O^{sc}	37.3	Salt bridge
K177-NH^{sc}	E409-O^{sc}	30.9	Salt bridge
K477-NH^{sc}	E559-O^{sc}	27.2	Salt bridge
K27-NH^{sc}	E259-O^{sc}	24.2	Salt bridge
K552-NH^{sc}	E634-O^{sc}	23.5	Salt bridge

K327-NH^{sc}	E559-O^{sc}	14.8	Salt bridge
K477-NH^{sc}	E709-O^{sc}	13.6	Salt bridge
K402-NH^{sc}	E334-O^{sc}	11.5	Salt bridge
K627-NH^{sc}	E709-O^{sc}	10.5	Salt bridge

Table S9: Intra-molecular H-bonds occupancies for the five PGGGQ residues of protofilament A, calculated over the course of the MD simulation on the 5O3L PHF dimer. Only occupancies above 5% were considered. The superscript BB indicates backbone atoms whereas superscript SC indicates side chain atoms.

Donor	***Acceptor***	***Occupancy (%)***	***Type***
**Protofilament A**			
G479-NH^{bb}	P328-O^{bb}	93.1	H-bond
G329-NH^{bb}	P178-O^{bb}	87.6	H-bond
G629-NH^{bb}	P478-O^{bb}	85.4	H-bond
Q332-NH^{bb}	G181-O^{bb}	85.1	H-bond
G179-NH^{bb}	P28-O^{bb}	83.7	H-bond
Q482-NH^{bb}	G331-O^{bb}	80.3	H-bond
Q632-NH^{bb}	G481-O^{bb}	76.3	H-bond
Q182-NH^{bb}	G31-O^{bb}	68.3	H-bond
Q182-NH^{sc}	Q332-O^{sc}	17.7	H-bond
Q332-NH^{sc}	Q482-O^{sc}	12.2	H-bond
Q482-NH^{sc}	Q632-O^{sc}	8.9	H-bond
Q32-NH^{sc}	Q182-O^{sc}	8.5	H-bond

Table S10: Intra-molecular H-bonds occupancies for the five PGGGQ residues of protofilament A calculated over the course of the MD simulation of protofilament A. Only occupancies above 5% were considered. The superscript BB indicates backbone atoms whereas superscript SC indicates side chain atoms.

Donor	***Acceptor***	***Occupancy (%)***	***Type***
**Protofilament A**			
G179-NH^{bb}	P103-O^{bb}	82.3	H-bond
G104-NH^{bb}	P28-O^{bb}	58.1	H-bond
G254-NH^{bb}	P178-O^{bb}	55.6	H-bond
G256-NH^{bb}	G330-O^{bb}	52.3	H-bond
Q107-NH^{bb}	G31-O^{bb}	52.0	H-bond
Q182-NH^{bb}	G106-O^{bb}	50.9	H-bond
Q257-NH^{bb}	G181-O^{bb}	42.8	H-bond
Q332-NH^{bb}	G256-O^{bb}	42.8	H-bond
G256-NH^{bb}	G330-O^{bb}	32.5	H-bond
G31-NH^{bb}	G105-O^{bb}	32.0	H-bond
G106-NH^{bb}	G180-O^{bb}	16.0	H-bond
G329-NH^{bb}	P253-O^{bb}	14.3	H-bond
Q32-NH^{sc}	Q107-O^{sc}	6.7	H-bond
Q107-NH^{sc}	Q182-O^{sc}	6.0	H-bond
G330-NH^{bb}	G254-O^{bb}	5.7	H-bond
Q257-NH^{sc}	Q332-O^{sc}	5.7	H-bond
Q182-NH^{sc}	Q257-O^{sc}	5.5	H-bond

Table S11: Intra-molecular H-bonds occupancies for the five PGGGQ residues of 7P65 protofilament calculated over the course of the MD simulation. Only occupancies above 5% were considered. The superscript BB indicates backbone atoms whereas superscript SC indicates side chain atoms.

Donor	***Acceptor***	***Occupancy (%)***	***Type***
	**Protofilament**		
Q66-NH^{bb}	G404-O^{bb}	34.45	H-bond
Q179-NH^{bb}	G65-O^{bb}	30.71	H-bond
Q292-NH^{bb}	G178-O^{bb}	28.74	H-bond
G289-NH^{bb}	P175-O^{bb}	27.83	H-bond
G176-NH^{bb}	P62-O^{bb}	27.50	H-bond
Q405-NH^{sc}	Q66-O^{sc}	25.41	H-bond
Q66-NH^{sc}	Q179-O^{sc}	24.37	H-bond
Q179-NH^{sc}	Q292-O^{sc}	19.28	H-bond
G63-NH^{bb}	P401-O^{bb}	18.09	H-bond
G64-NH^{bb}	G177-O^{bb}	11.64	H-bond
G291-NH^{bb}	G176-O^{bb}	10.8	H-bond
G290-NH^{bb}	P288-O^{bb}	9.38	H-bond
Q66-NH^{sc}	Q405-O^{sc}	8.29	H-bond
Q179-NH^{sc}	Q66-O^{sc}	7.91	H-bond
G291-NH^{bb}	G177-O^{bb}	7.42	H-bond
G178-NH^{bb}	G63-O^{bb}	7.12	H-bond
G403-NH^{bb}	G64-O^{bb}	6.75	H-bond
G178-NH^{bb}	G64-O^{bb}	6.55	H-bond
G404-NH^{bb}	G402-O^{bb}	5.1	H-bond

Table S12: Statistical analysis of the *glycine triads* Φ and ψ angles in the protofilament (7P65) – PHOX15 complex. The glycine Φ and ψ angles are scaled in the 0-360° range.

	Residue / angle	***Min angle***	***Max angle***	***Median angle***	***Mean angle***	***Std. deviation***	***Variance***
Filament 1	289 ϕ	56.96	329.20	268.82	256.00	34.55	1193.84
	289 Ψ	0.00	360.00	187.13	155.09	109.93	12083.74
	290 ϕ	26.75	353.58	195.84	193.87	86.49	7481.02
	290 Ψ	0.00	360.00	230.56	220.39	125.91	15854.11
	291 ϕ	14.31	336.23	273.15	229.41	84.80	7191.83
	291 Ψ	0.05	359.83	186.83	186.37	23.66	559.81
Filament 2	176 ϕ	72.54	311.23	238.74	229.12	38.78	1503.6
	176 Ψ	0.00	360.00	213.27	199.63	76.84	5904.45
	177 ϕ	25.56	339.58	266.96	214.56	88.2	7778.52
	177 Ψ	0.00	360,00	187.43	198.73	98.94	9789.37
	178 ϕ	15.82	339.00	221.23	183.71	98.96	9792.89
	178 Ψ	115.75	357.79	196.04	197.95	28.62	818.95
Filament 3	63 ϕ	66.3	319.29	209.49	217.86	39.68	1574.76
	63 Ψ	0.00	360,00	187.72	177.47	114.29	13061.51
	64 ϕ	28.41	344.07	234.53	202.27	82.82	6858.82
	64 Ψ	0.00	360.00	188.01	199.27	101.84	10371.43
	65 ϕ	13.52	332.19	179.62	174.23	94.07	8849.31
	65 Ψ	110.36	357.95	200.52	199.36	22.61	511.00
Filament 4	402 ϕ	31.96	357.3	270.39	248.81	49.83	2482.74
	402 Ψ	0.00	360.00	183.79	202.75	97.50	9505.73
	403 ϕ	26.62	343.67	272.43	214.12	90.58	8204.5
	403 Ψ	0.00	360.00	173.03	198.58	104.16	10850.00
	404 ϕ	21.71	332.84	263.54	202.77	97.05	9418.18
	404 Ψ	0.06	359.97	205.77	222.79	60.49	3659.43

Filament 5	515 ϕ	5.71	338.94	134.41	144.4	73.34	5378.72
	515 Ψ	0.01	359.98	176.67	179.76	57.24	3276.89
	516 ϕ	25.65	348.35	266.48	223.53	79.09	6255.96
	516 Ψ	0.00	360.00	189.01	199.61	100.49	10098.93
	517 ϕ	4.25	346.89	160.60	174.93	93.43	8729.71
	517 Ψ	0.02	359.99	191.40	197.29	56.35	3175.71

FIGURES S1-S22

Figure S1: Predicted 3D ROCS-based alignments of PHOX5, PHOX8, PHOX15 and PHOX20 with compounds CHEMBL1601285 (*Potency* = 891.3 nM), CHEMBL1315044 (*Potency* = 707.9 nM), CHEMBL1499585 (*Potency* = 5011.0 nM) and CHEMBL1514736 (*Potency* = 14124.4 nM), respectively (https://pubchem.ncbi.nlm.nih.gov/bioassay/1460).

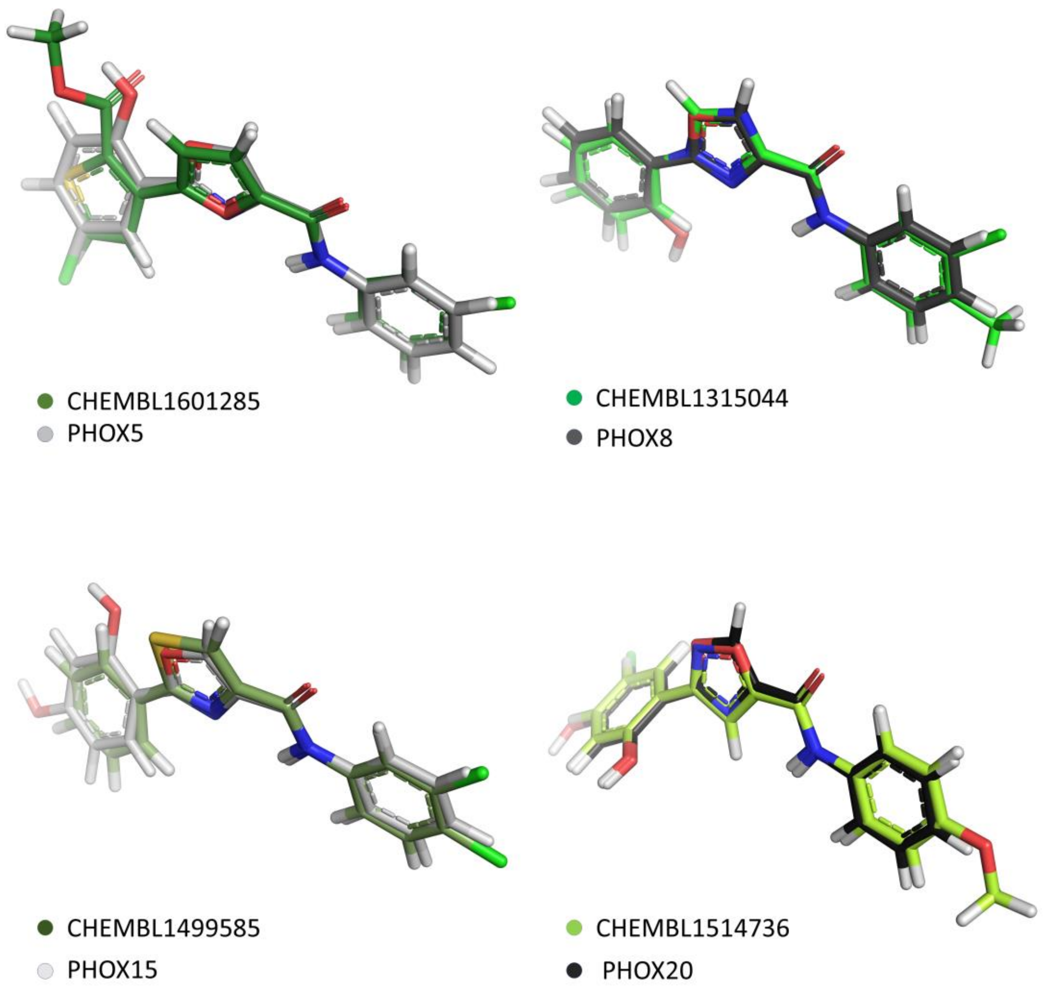

Figure S2: Comparison of the molecular descriptors of the PHOX compounds with respect to the 10th and 90th percentiles of the molecular properties of potent tau anti-aggregation ligands reported by [4].

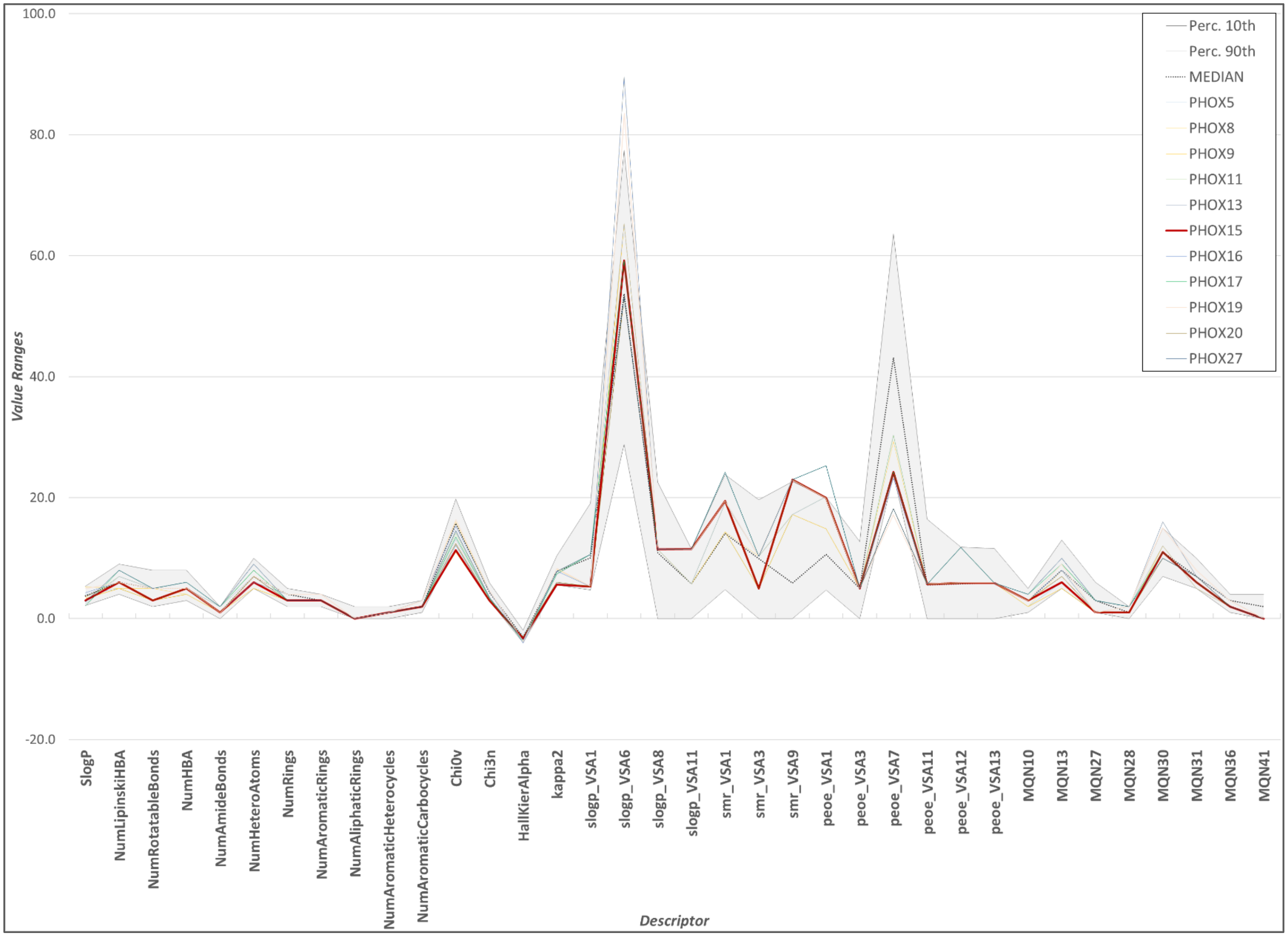

Figure S3: Toxicity and metabolic activity profiles of the PHOX compounds using a combined LDH- and MTT assays. Neuronally differentiated PC12 cells were exposed to the respective compounds. Each compound and concentration were measured in triplicates on two independent assay plates while mean±SEM of the two plates is shown. Statistical assessment of metabolic activity and cytotoxicity compared to carrier control was performed by One-way ANOVA using a Dunnet's post-hoc test. Statistically significant differences are indicated with an asterisk (* < 0.05, ** < 0.01, ***< 0.001). Source data are provided as a Source Data file; exact p-values are provided in the file.

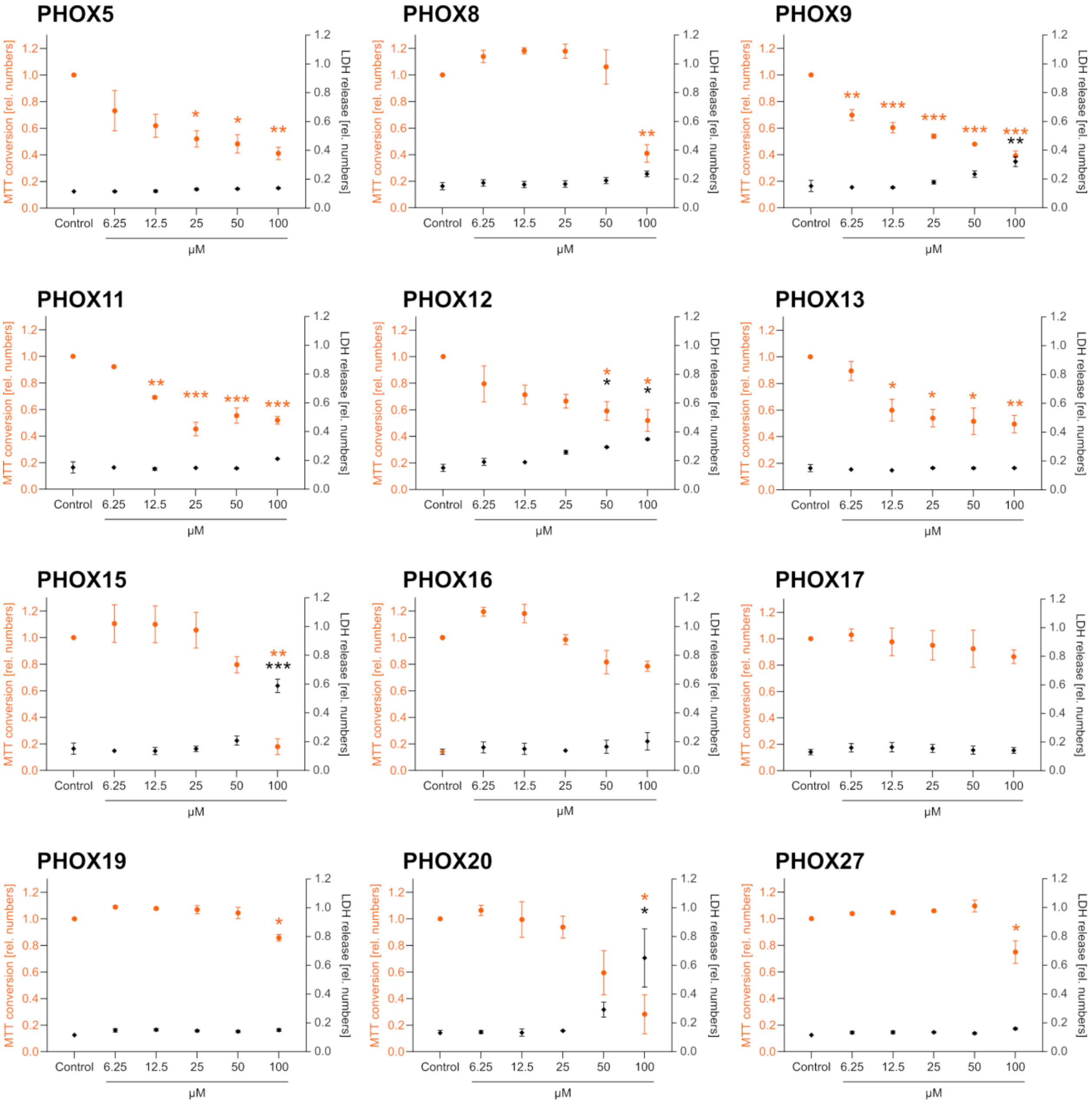

Figure S4: Volcano plot showing up- or down-regulated proteins in PHOX15 treated cultures compared to controls. All proteins showing significant up- or downregulation are indicated.

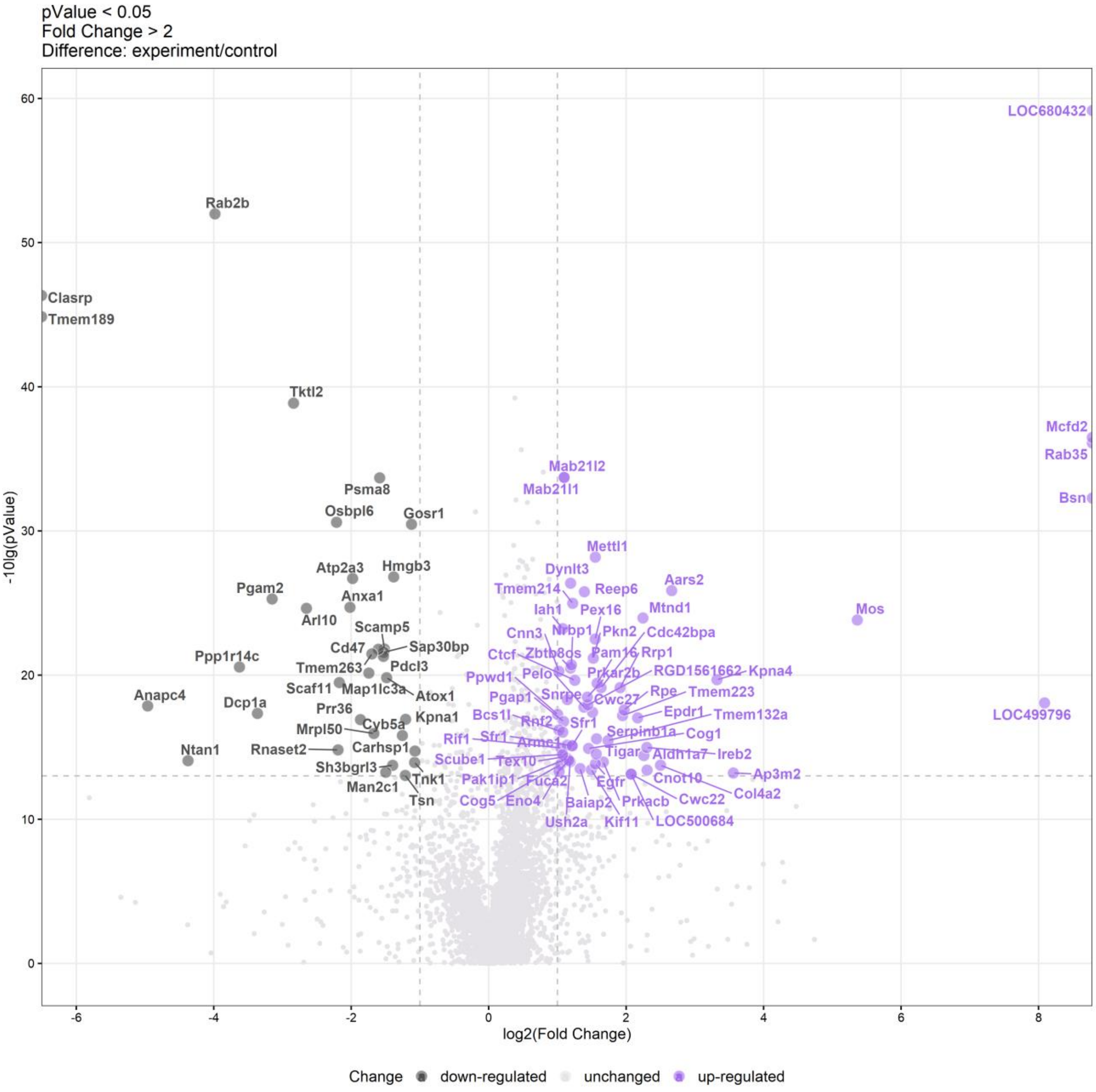

Figure S5: Coverage of the tau sequence by the phosphoproteomic analysis.

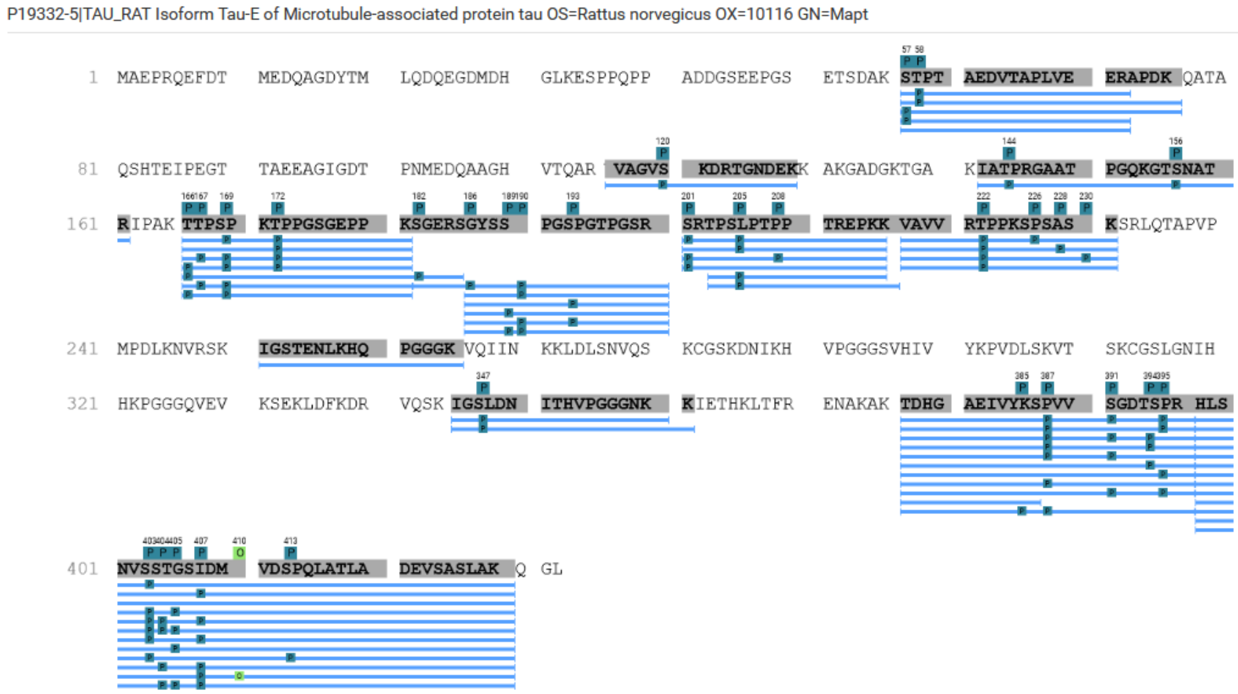

Figure S6: RMSD of the backbone atoms of protofilament A **(a)** and of the PHF dimer **(b)** over the MD simulation time on PDB structure 5O3L.

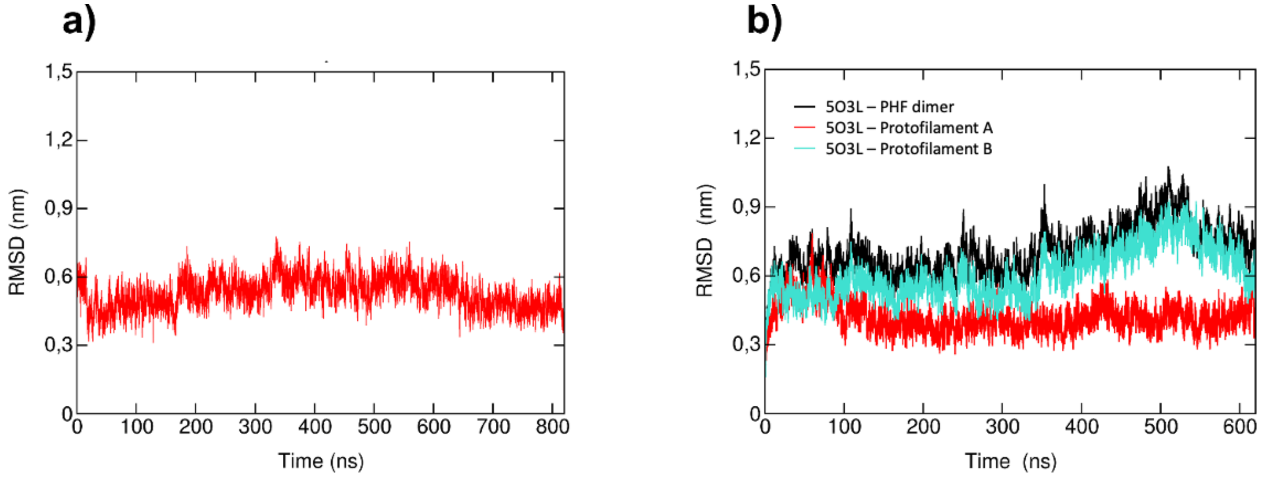

Figure S7: Radius of gyration of the protofilament A **(a)** and of the PHF dimer **(b)** over the MD simulation time on PDB structure 5O3L.

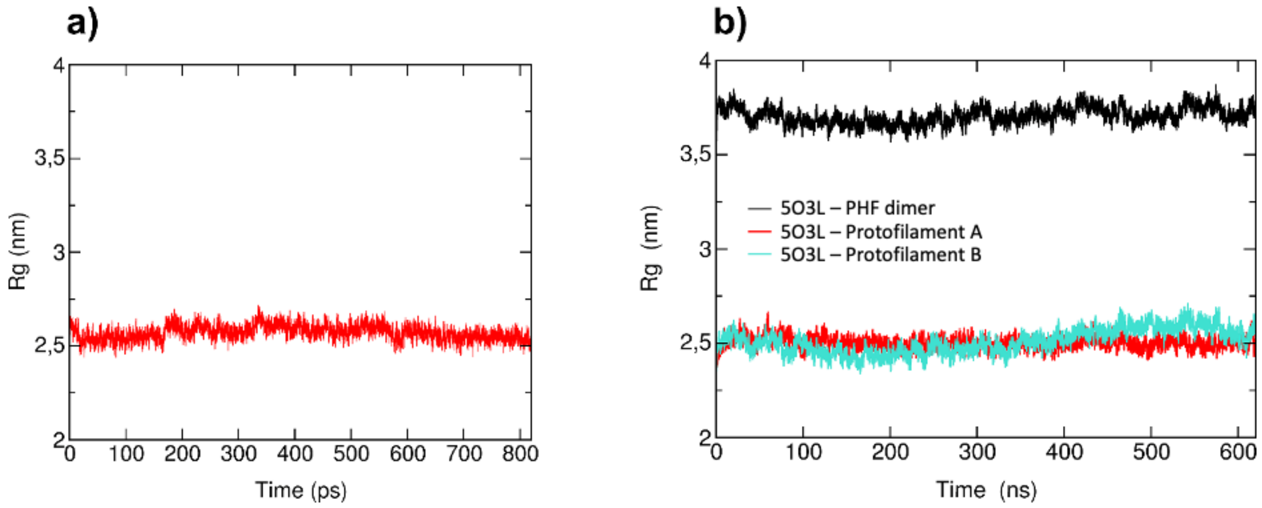

Figure S8: Secondary structure propensity of the protofilament A **(a)** and of the PHF dimer **(b)** during the MD simulation time on PDB structure 5O3L, calculated according to the DSSP formalism.

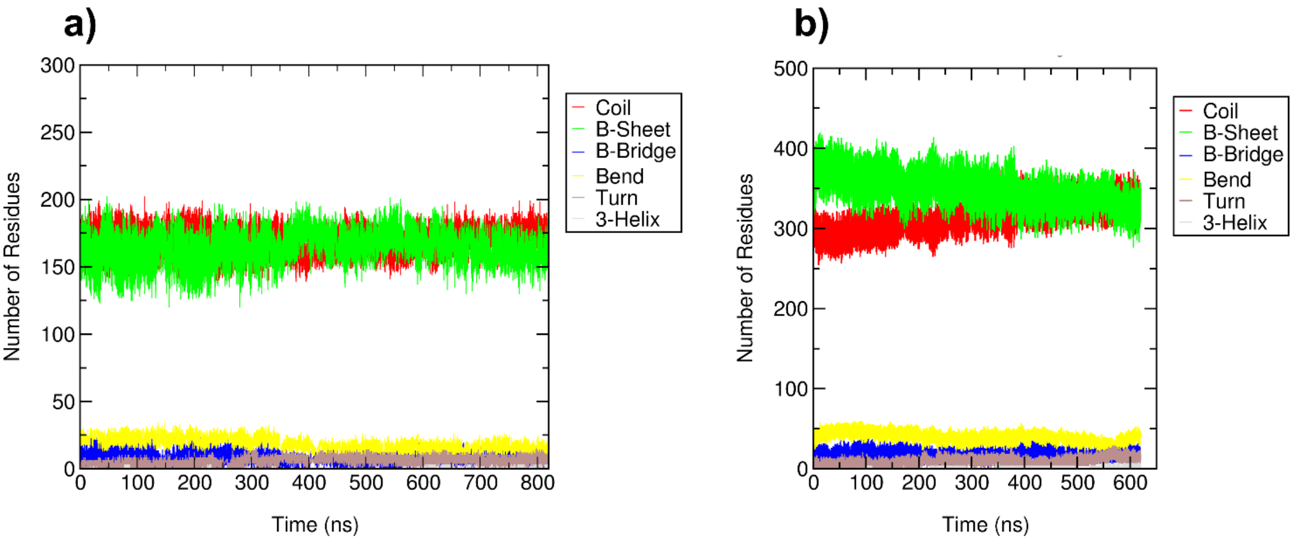

Figure S9: (**a**) Average intra-molecular H-bonds of the protofilament A during the MD simulation time on PDB structure 5O3L. (**b**) Average inter-molecular H-bonds of the PHF dimer (protofilament A-protofilament B) during the MD simulation time on PDB structure 5O3L. Standard deviation is shown as dotted lines. Curves are smoothed by reporting values of 1 every 10 of the collected MD frames.

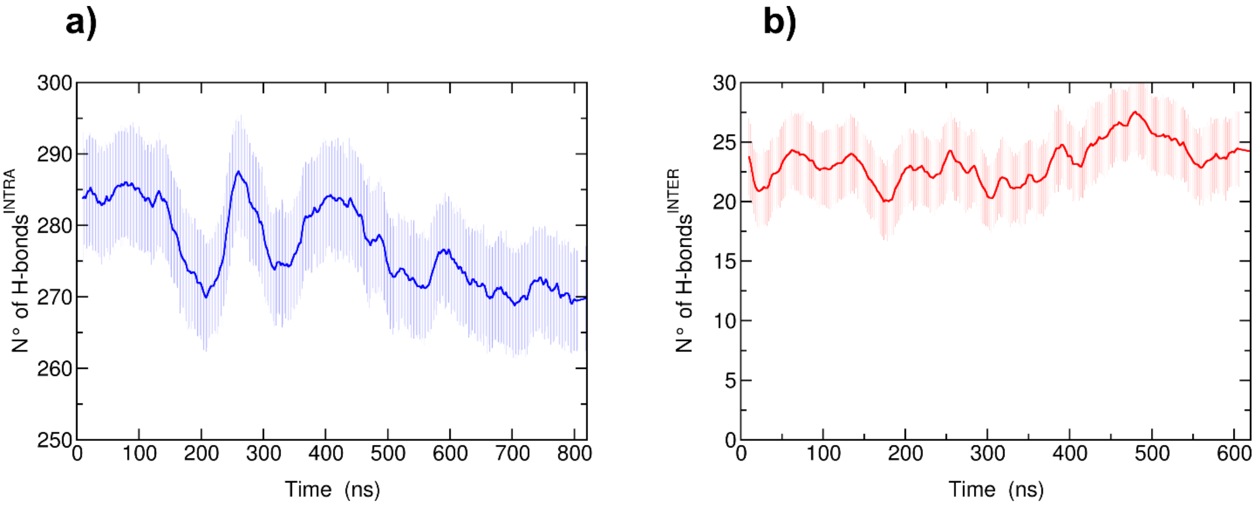

Figure S10: Correlation matrix showing Pearson correlation coefficients (P_c) for a number of protein and pockets descriptors. P1, P2 and P3 stand for pocket 1, pocket 2 and pocket 3 identified on PDB structure 5O3L.

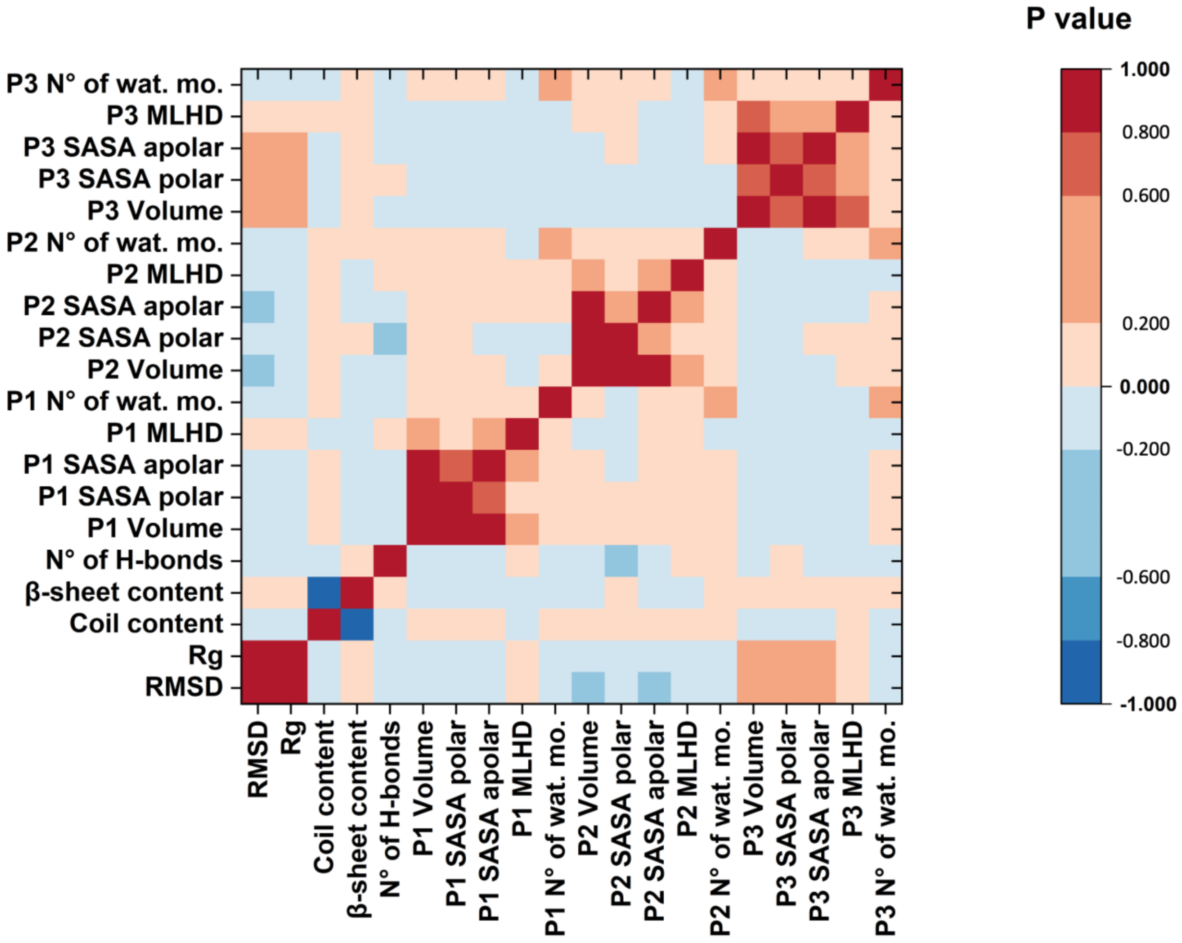

Note: N° of wat Mo. – number of water molecules; MLHD - mean local hydrophobic density; SASA apolar - solvent accessible surface area, apolar component; SASA polar - solvent accessible surface area, polar component; Volume – volume of the pocket; N° of H-bonds – number of hydrogen bonds interactions established by residues lining the pocket; b-sheet content – residues of the pocket framed into beta strands; Coil content – residues of the pocket framed into non-defined secondary structures; Rg – radius of gyration; RMSD – root mean square deviation.

Figure S11: Protofilament A (PDB structure: 5O3L) smoothed curves for pocket volume and surface accessible solvent area (SASA), plotted *versus* the MD simulation time. Curves are smoothed by reporting values of 1 every 10 of the collected MD frames. Standard deviation is shown as a black dotted line. **(a)** Pocket 1, **(b)** pocket 2, **(c)** pocket 3.

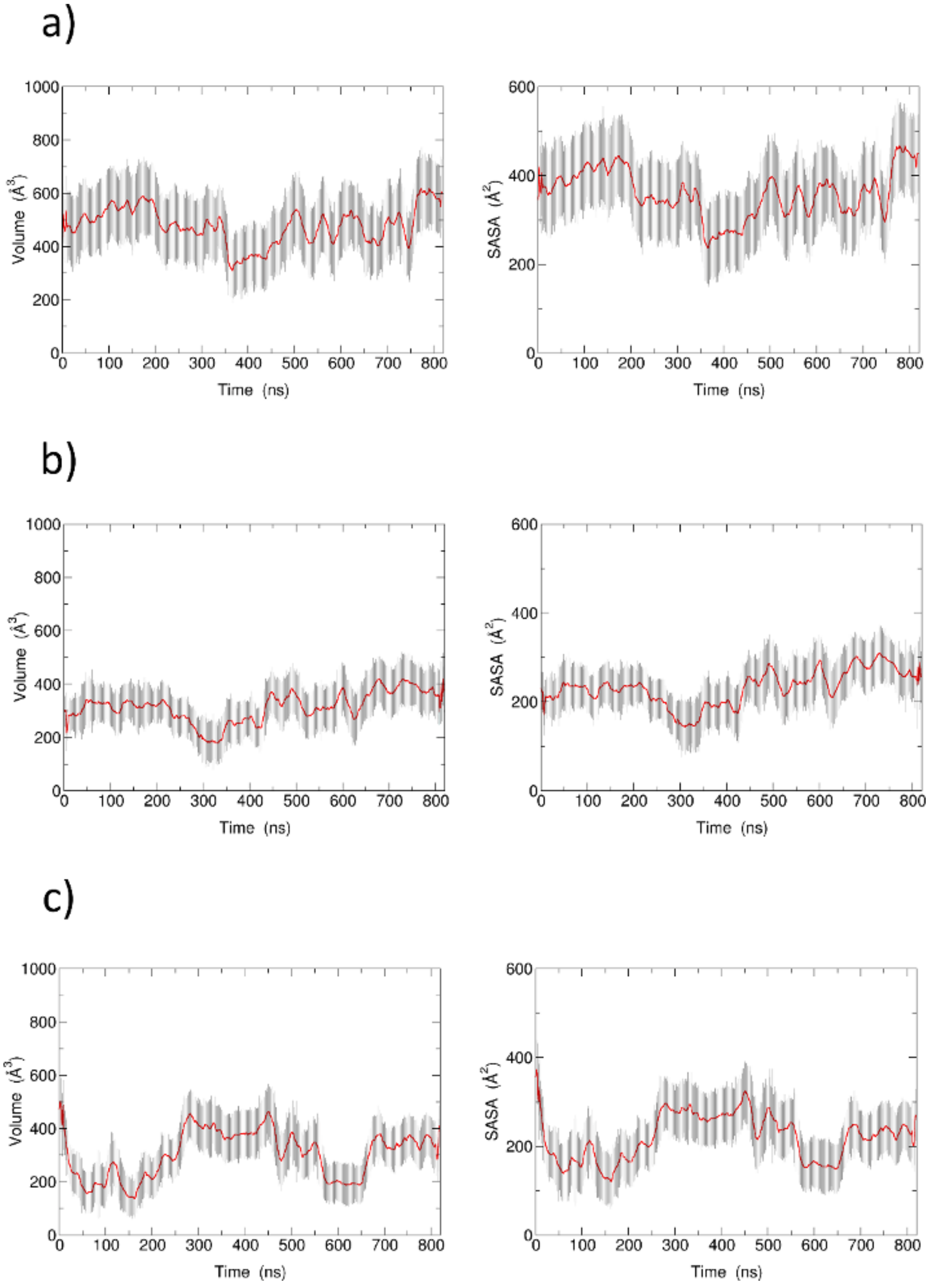

Figure S12: PHF dimer (PDB structure: 5O3L), protofilament A - smoothed curves for pocket volume and surface accessible solvent area (SASA) plotted versus the MD simulation time. Curves are smoothed by reporting values of 1 every 10 of the collected MD frames. Standard deviation is shown as a black dotted line. **(a)** Pocket 1, **(b)** pocket 2, **(c)** pocket 3.

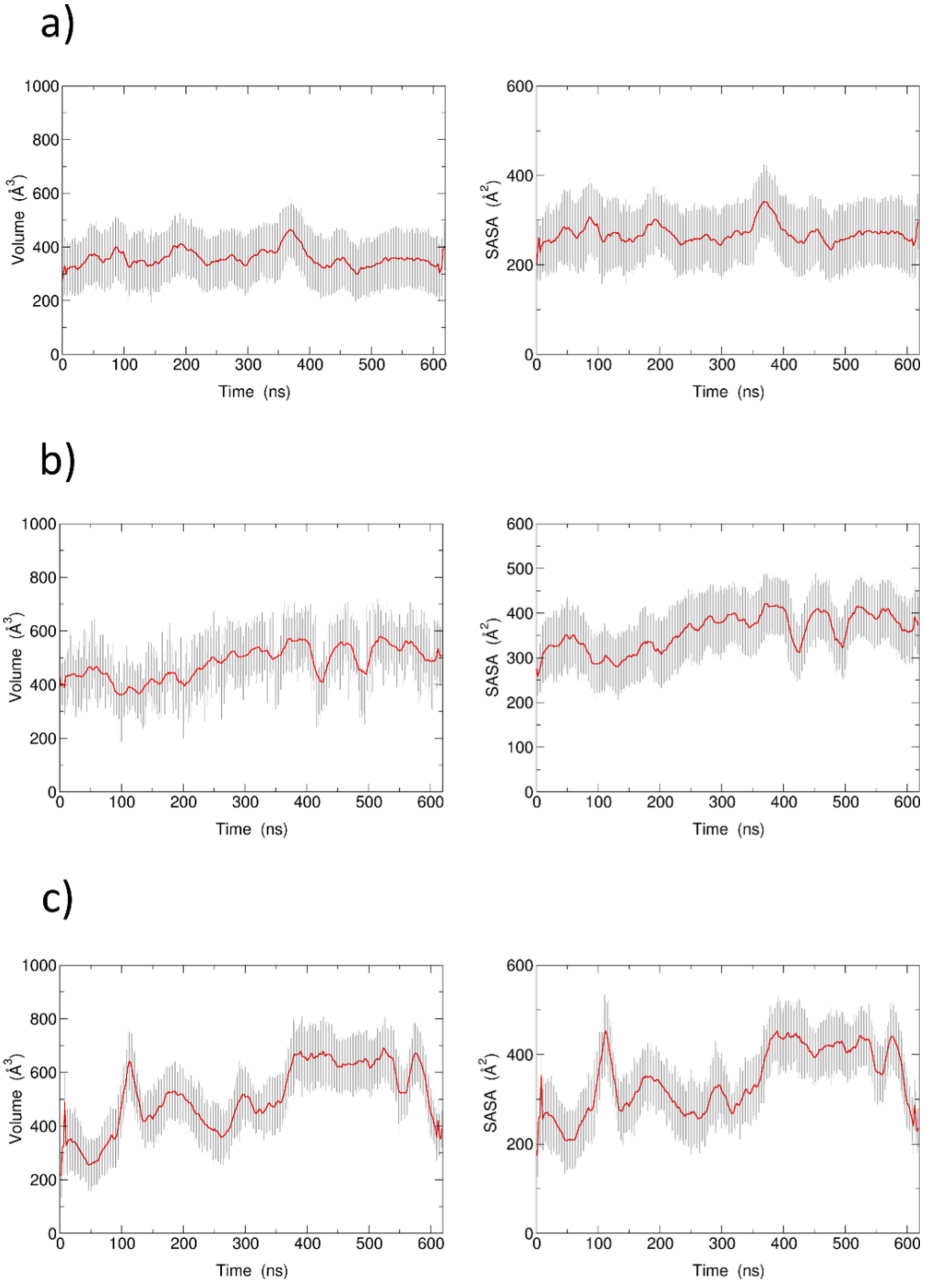

Figure S13: PHF dimer (PDB structure: 5O3L), protofilament B - smoothed curves for pocket volume and surface accessible solvent area (SASA) plotted versus the MD simulation time. Curves are smoothed by reporting values of 1 every 10 of the collected MD frames. Standard deviation is shown as a black dotted line. **(a)** Pocket 1*, **(b)** pocket 2*, **(c)** pocket 4* **(d)** pocket 3*.

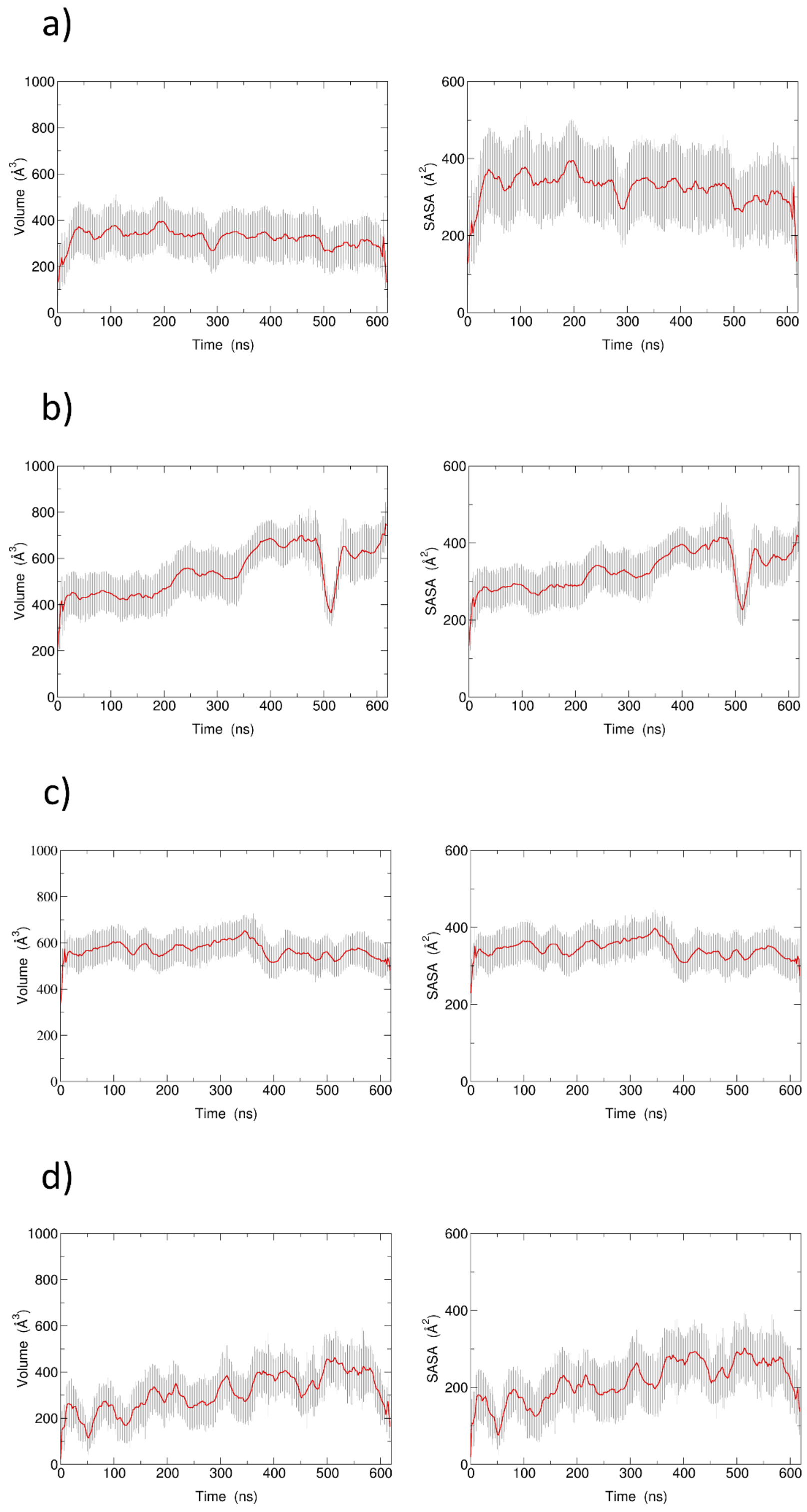

Figure S14: Ramachandran and heatmap plots showing the Φ and ψ angles of the glycine residues of the *glycine triads* of protofilament A (blue spots) and corresponding residues in the PHF dimer (orange spots) on PDB structure 5O3L. The glycine residues of the protofilament A showed significantly higher conformational variability compared to those of the same protofilament in the PHF dimer, which was also facilitated by the presence of a dense network of interactions along the dimerization interface of the two tau protofilaments.

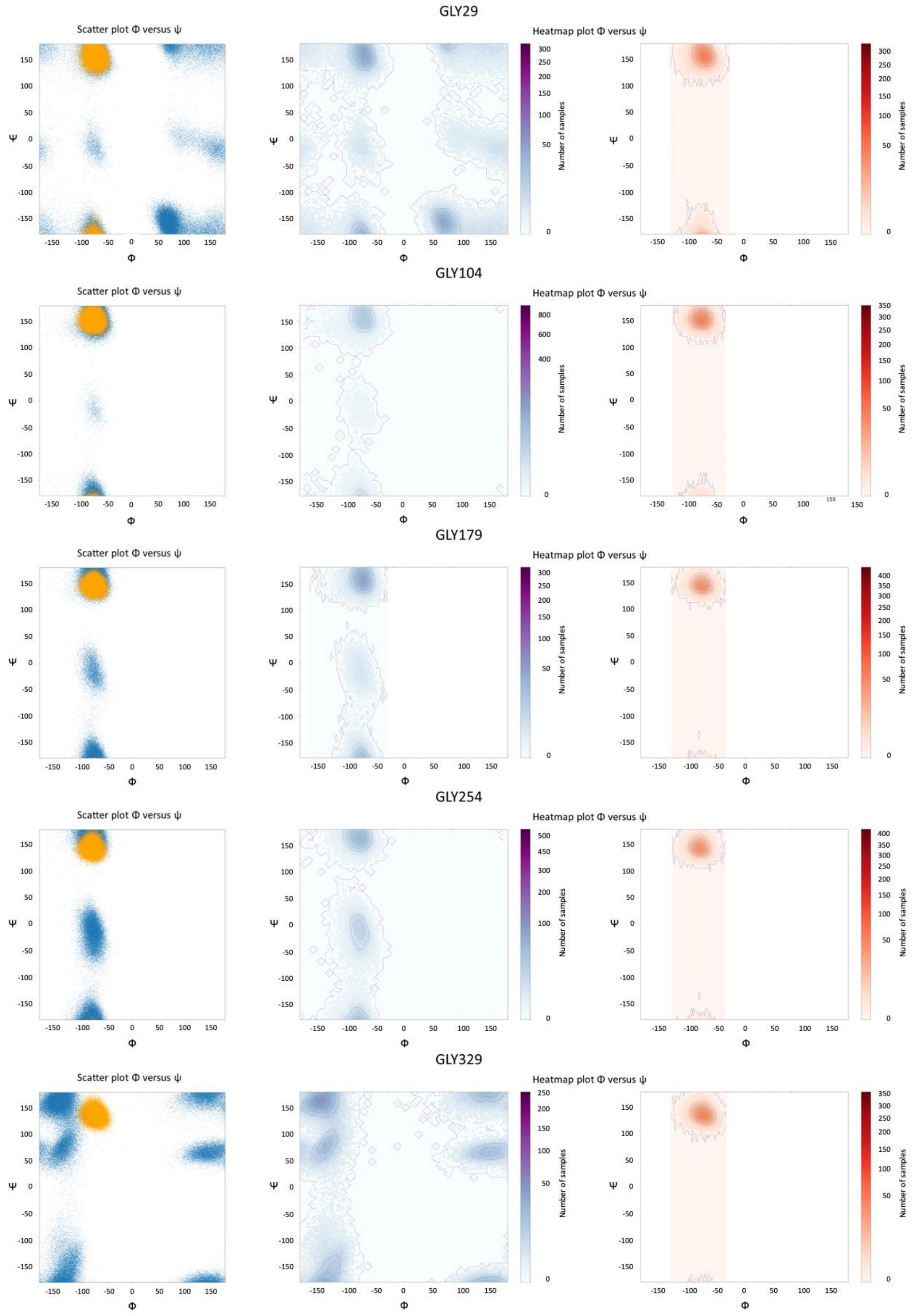

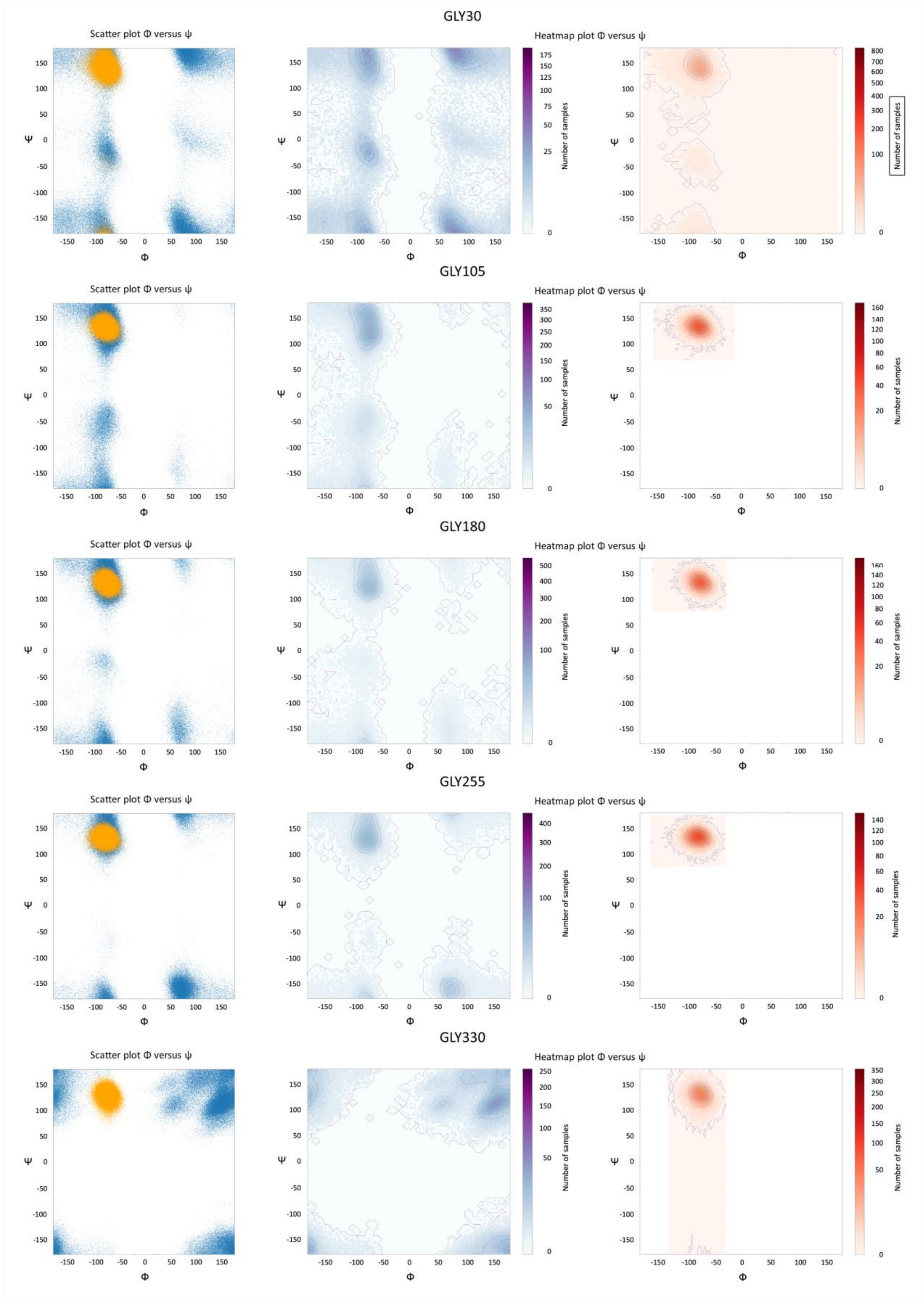
GLY30
Scatter plot Φ versus ψ
Heatmap plot Φ versus ψ
Number of samples
Φ
Ψ
GLY105
Scatter plot Φ versus ψ
Heatmap plot Φ versus ψ
Number of samples
GLY180
Scatter plot Φ versus ψ
Heatmap plot Φ versus ψ
Number of samples
GLY255
Scatter plot Φ versus ψ
Heatmap plot Φ versus ψ
Number of samples
GLY330
Scatter plot Φ versus ψ
Heatmap plot Φ versus ψ
Number of samples

GLY31

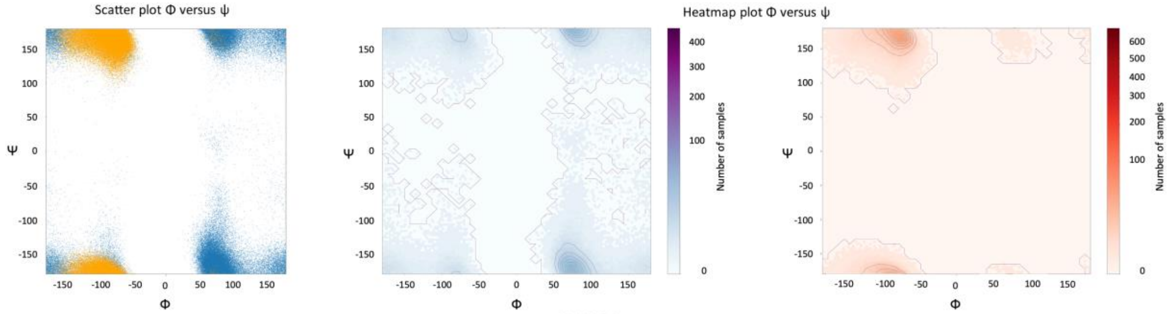

GLY106

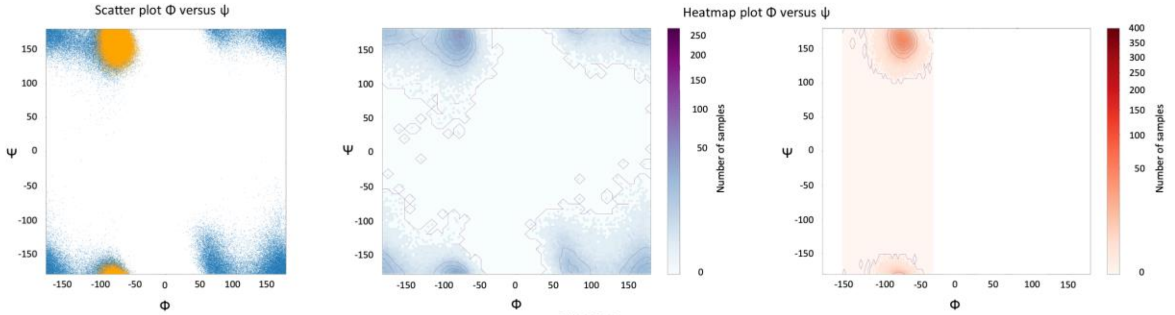

GLY181

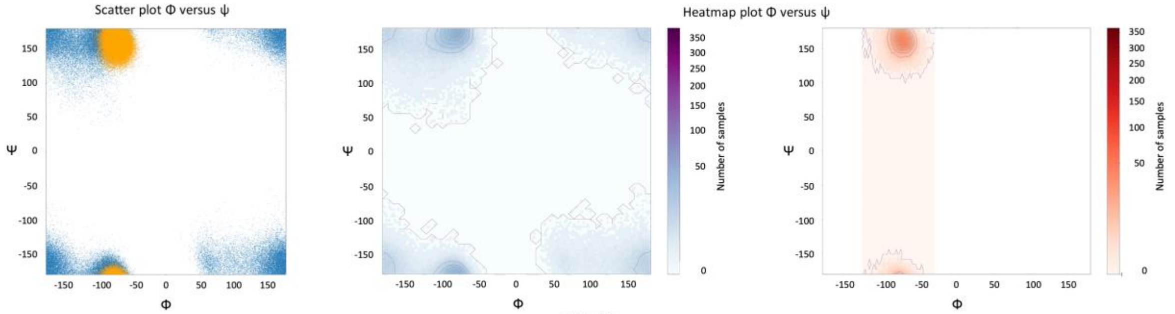

GLY256

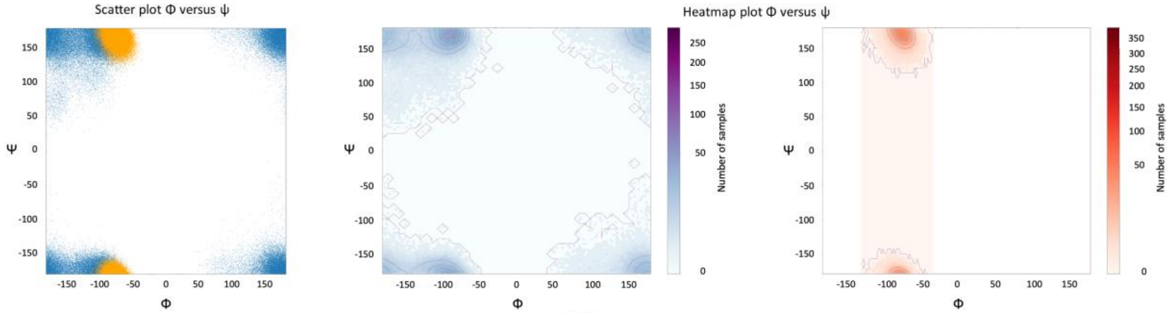

GLY331

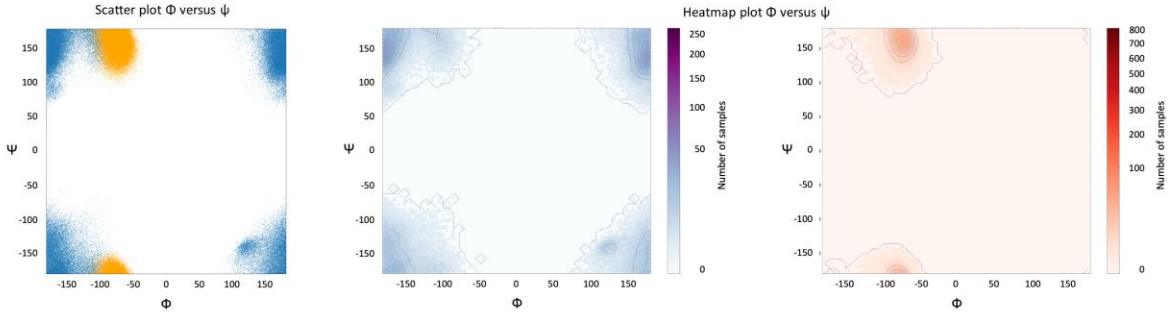

***Figure S15*:** Comparison of the MDpocket density maps on protofilament A and on PHF dimer of PDB structure 5O3L. Panels **a**) and **c**) report the results of the calculations with standard MDpocket parameters, while density maps shown in panels **b**) and **d**) are filtered by the druggable potential of the identified pockets.

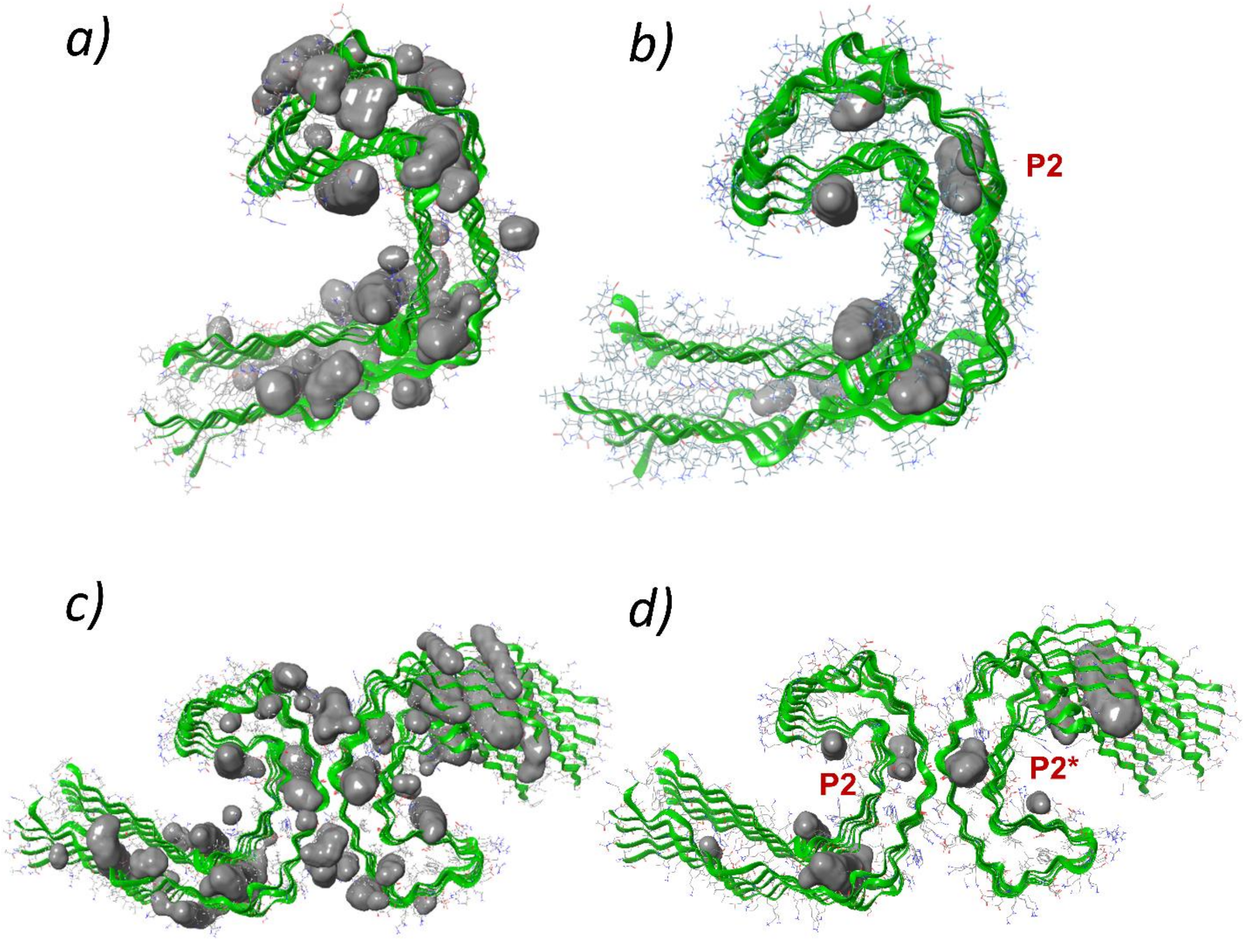

Figure S16: Pore radii determined by means of the HOLE program [22] conduced on PDB structure 5O3L. Curves are smoothed by reporting values of 1 every 10 of the collected MD frames. **(a)** Radius of pocket 2 from the MD simulation of protofilament A. **(b)** Radius of pocket 2 from the MD simulation of the PHF dimer. **(c)** Radius of pocket 2* from the MD simulation of the PHF dimer.

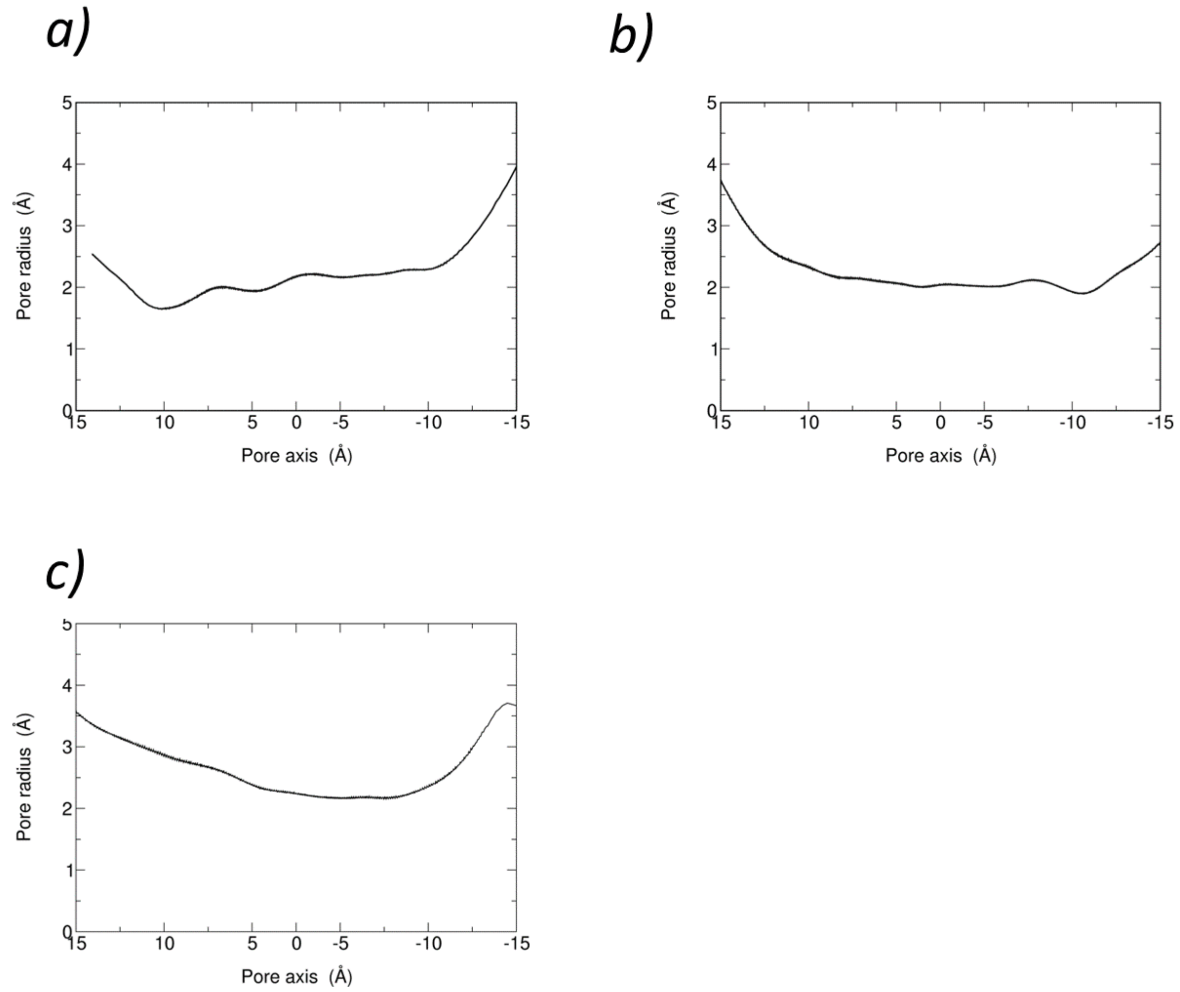

Figure S17: Pockets mapped on the structure of the PHF dimer (PDB ID: 5O3L). The novel pockets identified in this work are marked by red boxes. Yellow, grey and orange filled spheres highlight external pockets that were proposed by [11,26,27], respectively. Blue filled triangles denote binding pockets near the oligomer dimerization site proposed by [28].

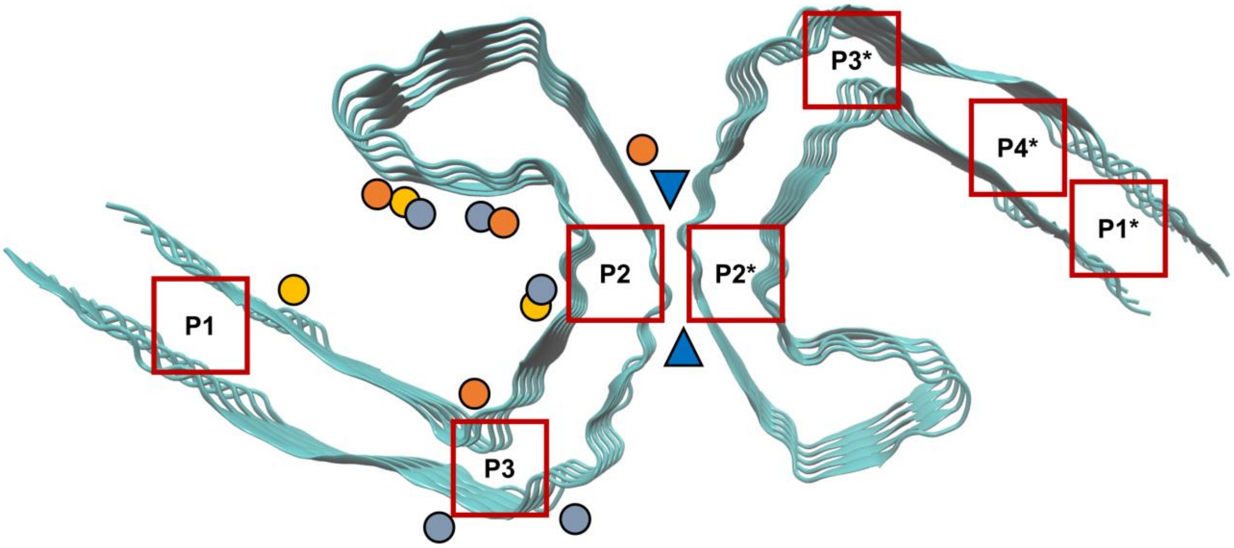

Figure S18: Radius of gyration **(panel A**) and RMSD plots (**panels B to D**) of the 7P65 protofilament over the MD simulation time. In particular, panel B reports the RMSD plot evaluated on the residues' backbone of the overall 7P65 structure. **Panel C** reports the RMSD plot evaluated on the backbone of residues lining the central pocket shown in Figure 5A, right (*i.e.*, filament 1 (residue identifiers: 272 to 283 and 294 to 299); filament 2 (residue identifiers: 159 to 170 and 181 to 186); filament 3 (residue identifiers: 46 to 57 and 68 to 73); filament 4 (residue identifiers: 385 to 396 and 407 to 412); filament 5 (residue identifiers: 498 to 509 and 520 to 525)). **Panel D** reports the RMSD plot evaluated on the backbone of residues from PGGGQ segments (i.e., filament 1 (residue identifiers: 288 to 292); filament 2 (residue identifiers: 175 to 179); filament 3 (residue identifiers: 62 to 66); filament 4 (residue identifiers: 401 to 405); filament 5 (residue identifiers: 514 to 518)). Curves are smoothed by reporting values of 1 every 10 of the collected MD frames.

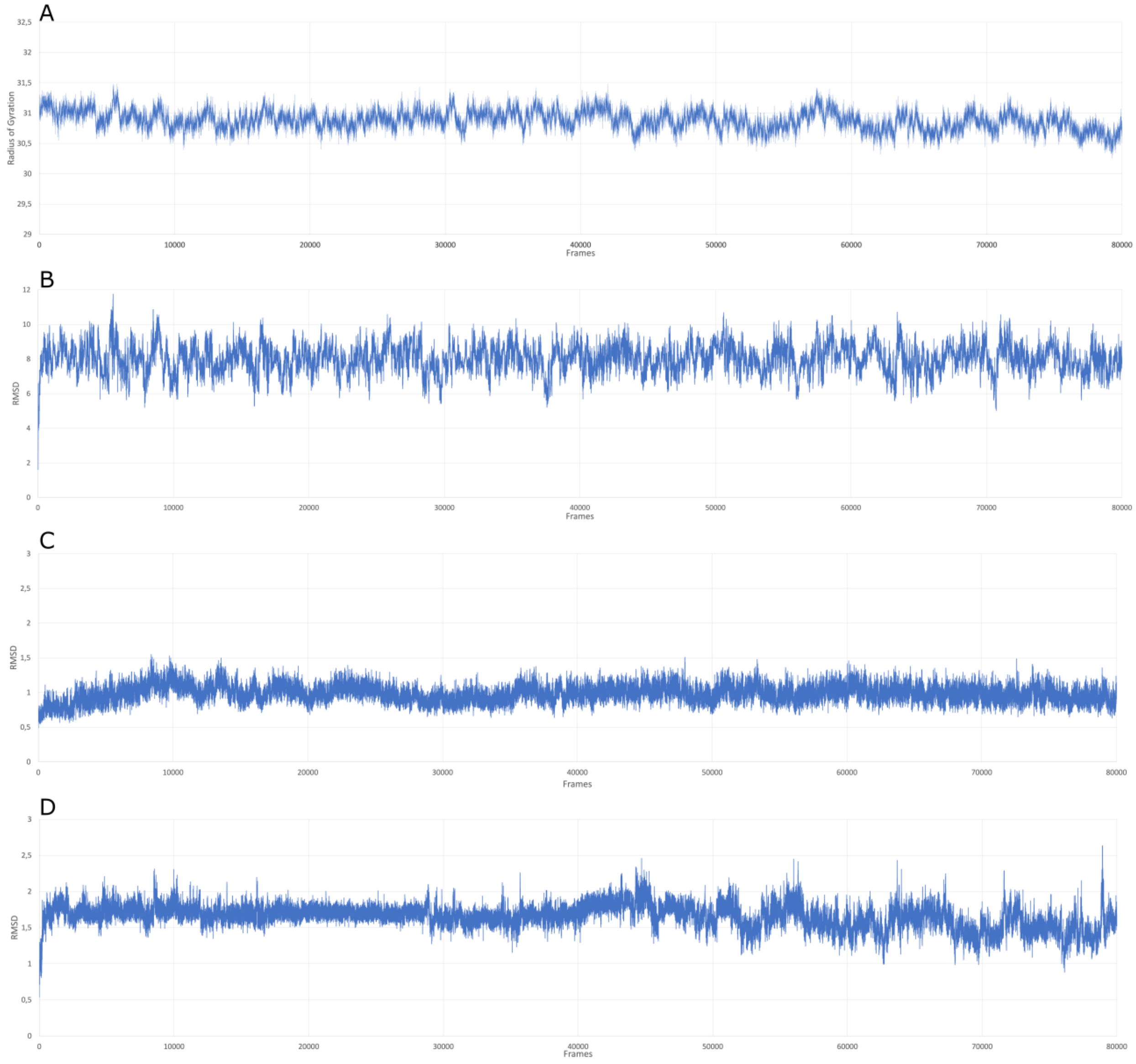

Figure S19: Schematic representation of non-covalent interactions between the protofilaments A and B at the PHF dimerization interface (5O3L cryo-EM structure). **A)** 3D representation of the H-bond interactions occurring between the glycine triads. **B)** Representation of the H-bonds network established by glycines across the different filaments of tau at the PHF interface.

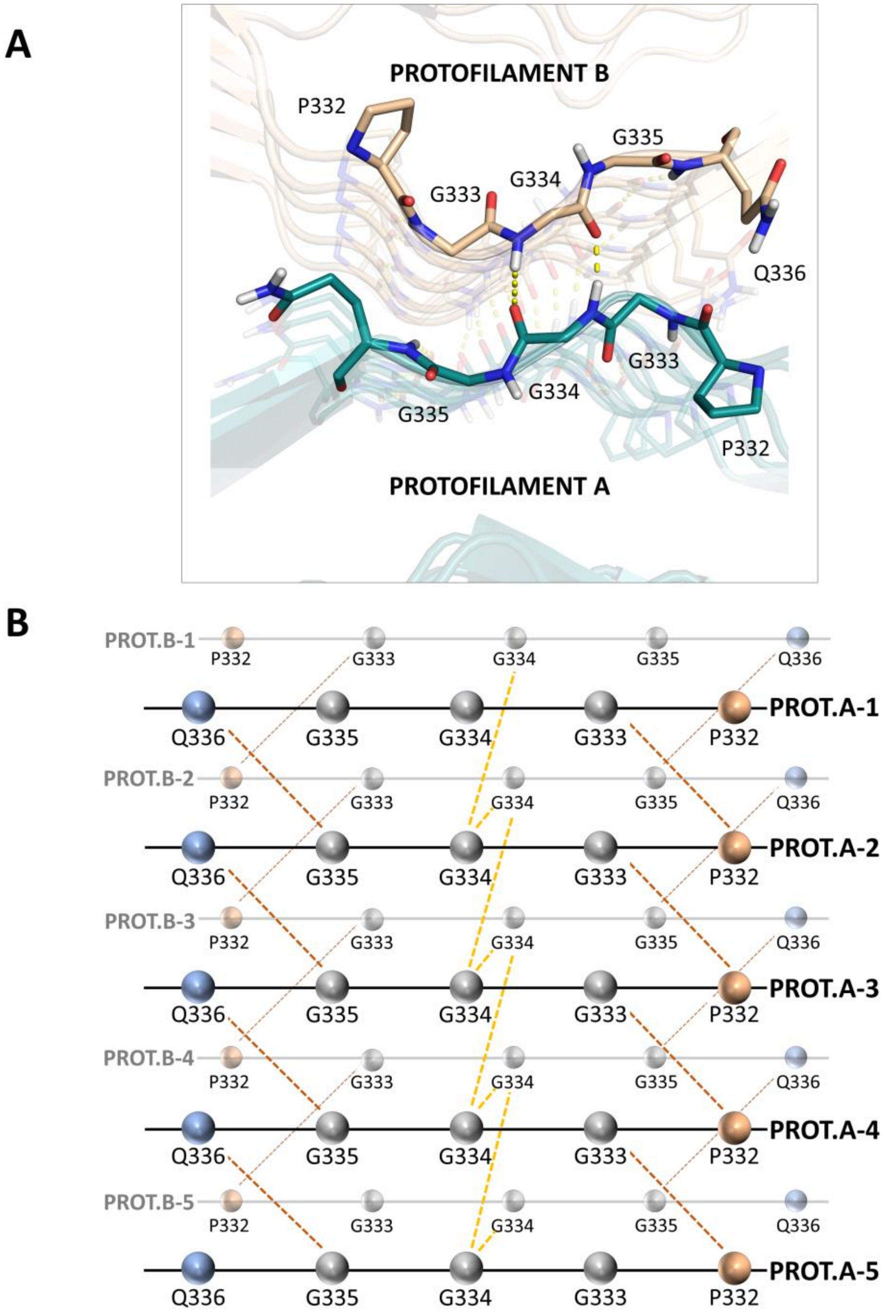

Figure S20: Predicted binding mode of compound PHOX15 into pocket 2 of 5O3L (**panel A**) and into the central channel-like pocket of 7P65 (**panel B**). According to the performed calculations, PHOX15 establishes H-bond interactions with residues Gly104, Gly179, Gly201 and Gly255. Moreover, hydrophobic contacts were observed between the phenyl rings of PHOX15 and the side chains of Leu128, Leu203 and Leu278 (residues numbering refers to **Table A** of the Supporting Information). The same calculations into the Px channel-like pocket of 7P65 showed the resorcinol moiety of PHOX15 accommodating in proximity of the fourth filament and establishing H-bond interactions with residues Ser324 (fifth filament) and Asn327 (fourth filament). While the oxygen atom of isoxazole moiety and the carbonyl group of the amide were predicted to perform H-bond interactions with the side chains of residues Ser324 (fourth filament) and Ser324 (third filament), respectively (**panel B**).

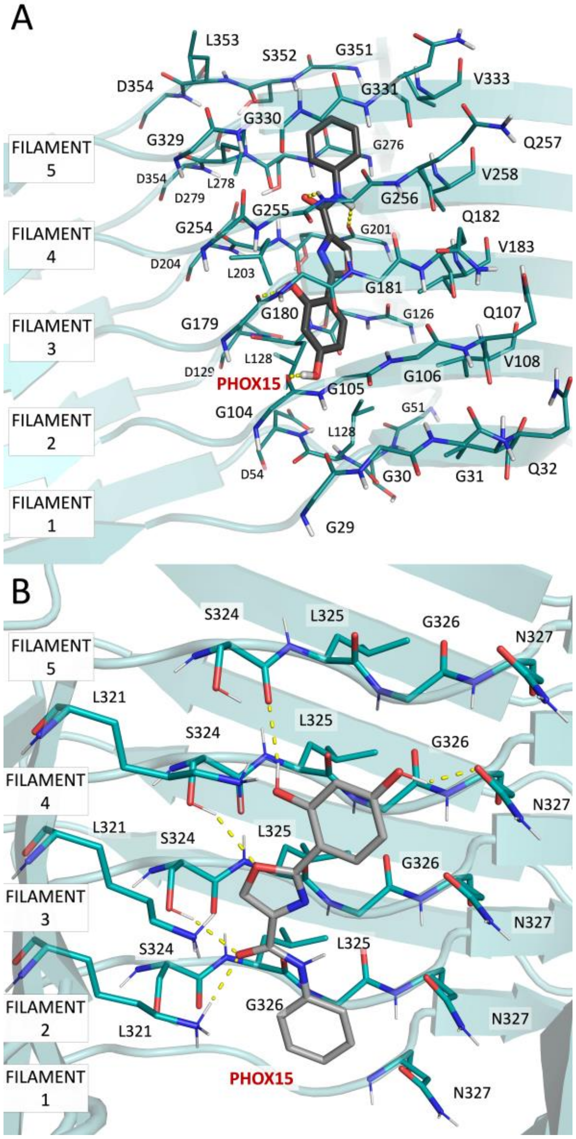

Figure S21: Ramachandran plots showing the Φ and ψ of the *glycines triads* of the cryptic pocket P2 in the complex protofilament A (5O3L) - PHOX15.

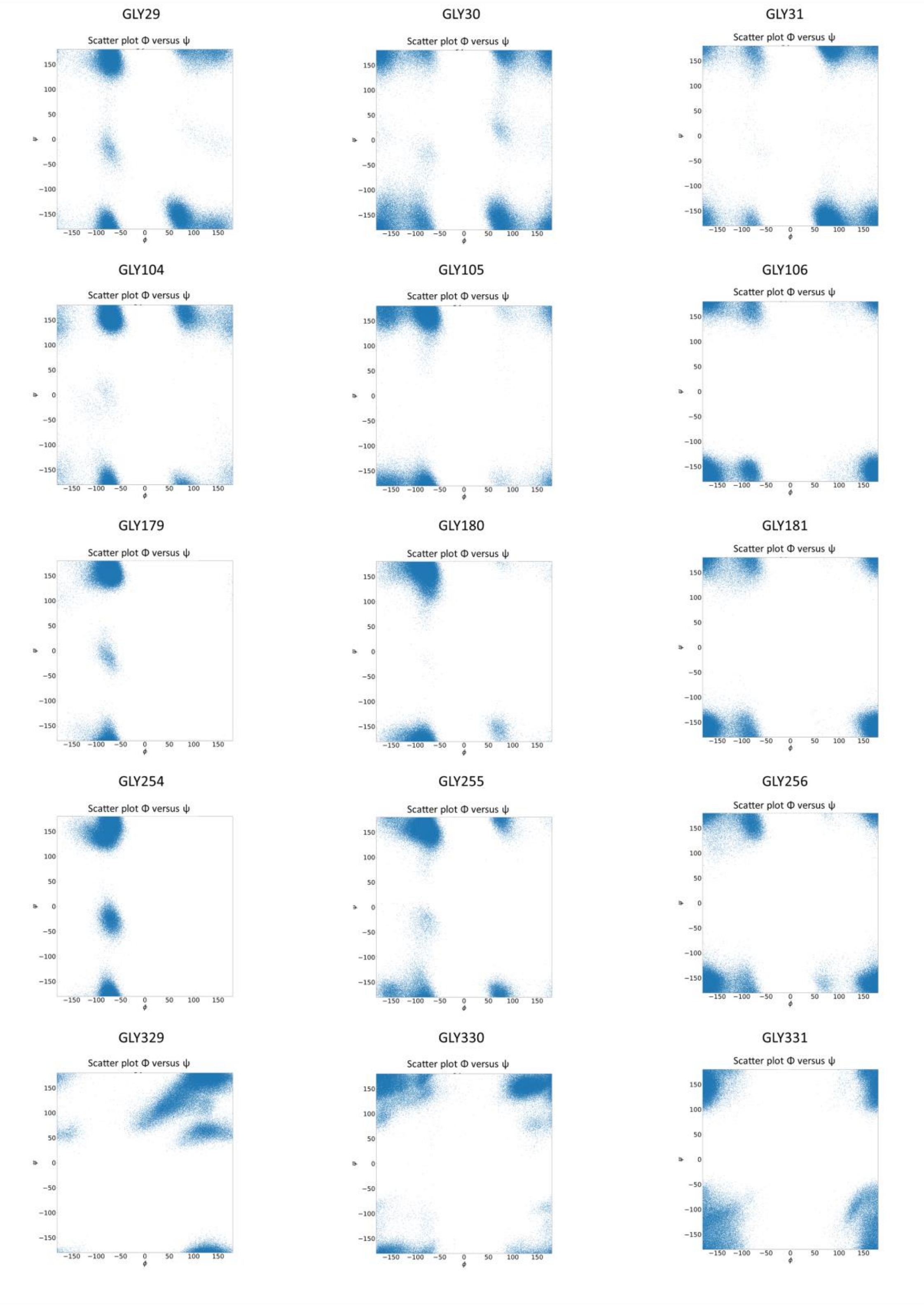

Figure S22: Radius of gyration (**panel A**) and RMSD plots (**panels B to E**) of the 7P65 protofilament in complex with PHOX15 over the MD simulation time. In particular, panel B reports the RMSD plot evaluated on the residues' backbone of the overall 7P65 structure. Panel C reports the RMSD plot evaluated on the backbone of residues lining the central pocket shown in Figure 5A, right (*i.e.*, filament 1 (residue identifiers: 272 to 283 and 294 to 299); filament 2 (residue identifiers: 159 to 170 and 181 to 186); filament 3 (residue identifiers: 46 to 57 and 68 to 73); filament 4 (residue identifiers: 385 to 396 and 407 to 412); filament 5 (residue identifiers: 498 to 509 and 520 to 525)). Panel D reports the RMSD plot evaluated on the backbone of residues from PGGGQ segments (*i.e.*, filament 1 (residue identifiers: 288 to 292); filament 2 (residue identifiers: 175 to 179); filament 3 (residue identifiers: 62 to 66); filament 4 (residue identifiers: 401 to 405); filament 5 (residue identifiers: 514 to 518)). Panel E reports the RMSD plot evaluated on the heavy atoms of PHOX15.

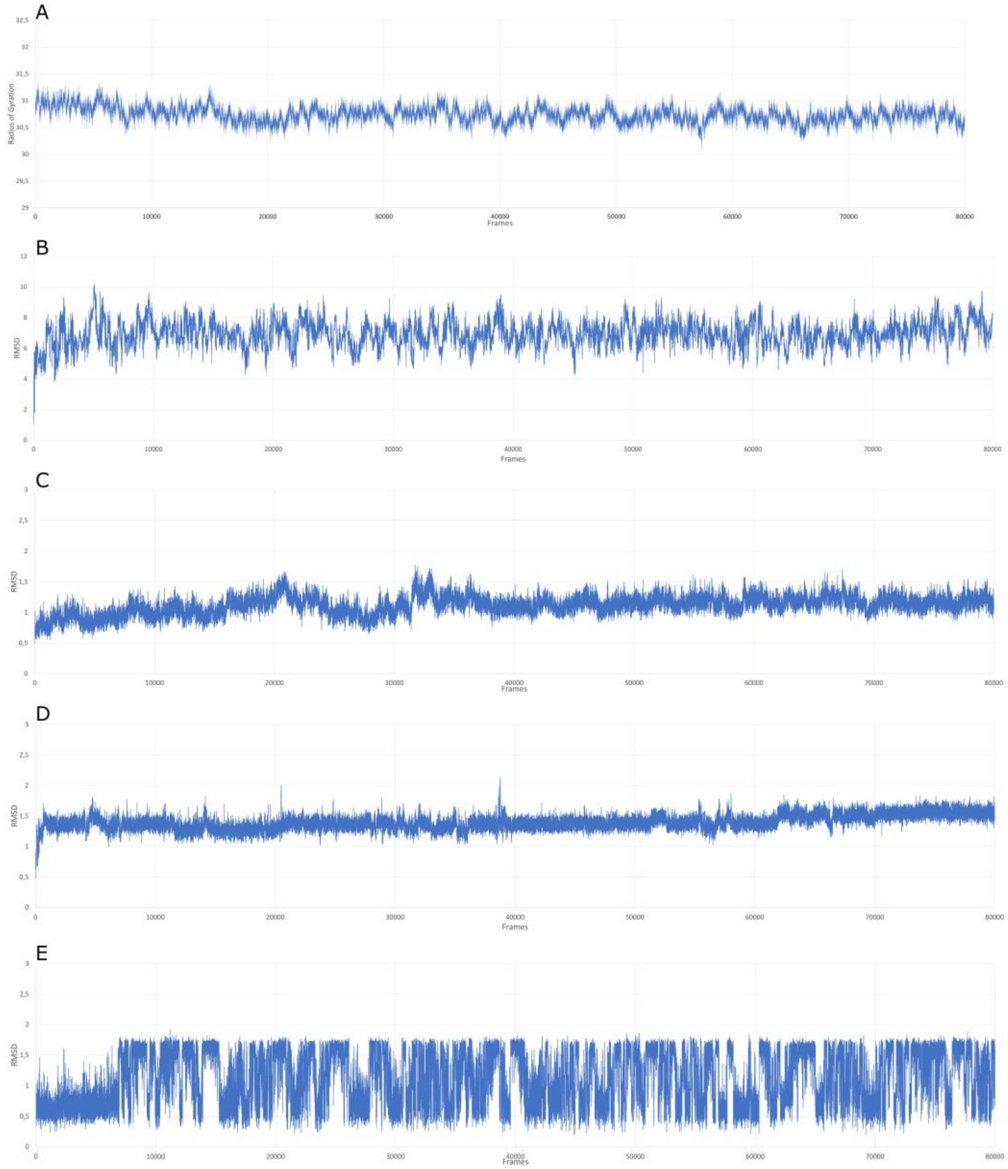

REFERENCES:

1 Gaulton, A. *et al.* The ChEMBL database in 2017. *Nucleic acids research* **45**, D945-D954, doi:10.1093/nar/gkw1074 (2017).

2 Hawkins, P. C., Skillman, A. G., Warren, G. L., Ellingson, B. A. & Stahl, M. T. Conformer generation with OMEGA: algorithm and validation using high quality structures from the Protein Databank and Cambridge Structural Database. *J Chem Inf Model* **50**, 572-584, doi:10.1021/ci100031x (2010).

3 Hawkins, P. C., Skillman, A. G. & Nicholls, A. Comparison of shape-matching and docking as virtual screening tools. *J Med Chem* **50**, 74-82, doi:10.1021/jm0603365 (2007).

4 Pinzi, L., Tinivella, A. & Rastelli, G. Chemoinformatics Analyses of Tau Ligands Reveal Key Molecular Requirements for the Identification of Potential Drug Candidates against Tauopathies. *Molecules* **26**, doi:10.3390/molecules26165039 (2021).

5 Pinzi, L. & Rastelli, G. Identification of Target Associations for Polypharmacology from Analysis of Crystallographic Ligands of the Protein Data Bank. *J Chem Inf Model* **60**, 372-390, doi:10.1021/acs.jcim.9b00821 (2020).

6 Fitzpatrick, A. W. P. *et al.* Cryo-EM structures of tau filaments from Alzheimer's disease. *Nature* **547**, 185-190, doi:10.1038/nature23002 (2017).

7 Tian, C. *et al.* ff19SB: Amino-Acid-Specific Protein Backbone Parameters Trained against Quantum Mechanics Energy Surfaces in Solution. *J Chem Theory Comput* **16**, 528-552, doi:10.1021/acs.jctc.9b00591 (2020).

8 Ryckaert, J.-P., Ciccotti, G. & Berendsen, H. J. C. Numerical integration of the cartesian equations of motion of a system with constraints: molecular dynamics of n-alkanes. *Journal of Computational Physics* **23**, 327-341, doi:10.1016/0021-9991(77)90098-5 (1977).

9 He, X., Man, V. H., Yang, W., Lee, T. S. & Wang, J. A fast and high-quality charge model for the next generation general AMBER force field. *J Chem Phys* **153**, 114502, doi:10.1063/5.0019056 (2020).

10 Jakalian, A., Bush, B. L., Jack, D. B. & Bayly, C. I. Fast, efficient generation of high-quality atomic charges. AM1-BCC model: I. Method. *J. Comput. Chem.* **21**, 132-146 (2000).

11 Shi, Y. *et al.* Cryo-EM structures of tau filaments from Alzheimer's disease with PET ligand APN-1607. *Acta neuropathologica* **141**, 697-708, doi:10.1007/s00401-021-02294-3 (2021).

12 Abraham, M. J. *et al.* GROMACS: High performance molecular simulations through multi-level parallelism from laptops to supercomputers. *Software X* **1–2**, 19-25 (2015).

13 Van Der Spoel, D. *et al.* GROMACS: fast, flexible, and free. *J Comput Chem* **26**, 1701-1718, doi:10.1002/jcc.20291 (2005).

14 McGibbon, R. T. *et al.* MDTraj: A Modern Open Library for the Analysis of Molecular Dynamics Trajectories. *Biophysical journal* **109**, 1528-1532, doi:10.1016/j.bpj.2015.08.015 (2015).

15 Sousa da Silva, A. W. & Vranken, W. F. ACPYPE - AnteChamber PYthon Parser interfacE. *BMC Res Notes* **5**, 367, doi:10.1186/1756-0500-5-367 (2012).

16 Bakan, A., Meireles, L. M. & Bahar, I. ProDy: protein dynamics inferred from theory and experiments. *Bioinformatics (Oxford, England)* **27**, 1575-1577, doi:10.1093/bioinformatics/btr168 (2011).

17 Humphrey, W., Dalke, A. & Schulten, K. VMD: visual molecular dynamics. *J Mol Graph* **14**, 33-38, 27-38, doi:10.1016/0263-7855(96)00018-5 (1996).
18 Pettersen, E. F. *et al.* UCSF Chimera--a visualization system for exploratory research and analysis. *J Comput Chem* **25**, 1605-1612, doi:10.1002/jcc.20084 (2004).
19 Schmidtke, P., Bidon-Chanal, A., Luque, F. J. & Barril, X. MDpocket: open-source cavity detection and characterization on molecular dynamics trajectories. *Bioinformatics (Oxford, England)* **27**, 3276-3285, doi:10.1093/bioinformatics/btr550 (2011).
20 Schmidtke, P., Le Guilloux, V., Maupetit, J. & Tuffery, P. fpocket: online tools for protein ensemble pocket detection and tracking. *Nucleic acids research* **38**, W582-589, doi:10.1093/nar/gkq383 (2010).
21 Schmidtke, P. & Barril, X. Understanding and predicting druggability. A high-throughput method for detection of drug binding sites. *J Med Chem* **53**, 5858-5867, doi:10.1021/jm100574m (2010).
22 Smart, O. S., Neduvelil, J. G., Wang, X., Wallace, B. A. & Sansom, M. S. HOLE: a program for the analysis of the pore dimensions of ion channel structural models. *J Mol Graph* **14**, 354-360, 376, doi:10.1016/s0263-7855(97)00009-x (1996).
23 Gentile, G. *et al.* 5-Aryl-4-carboxamide-1,3-oxazoles: potent and selective GSK-3 inhibitors. *Bioorg Med Chem Lett* **22**, 1989-1994, doi:10.1016/j.bmcl.2012.01.034 (2012).
24 Malmstrom, J. *et al.* Synthesis and structure-activity relationship of 4-(1,3-benzothiazol-2-yl)-thiophene-2-sulfonamides as cyclin-dependent kinase 5 (cdk5)/p25 inhibitors. *Bioorg Med Chem Lett* **22**, 5919-5923, doi:10.1016/j.bmcl.2012.07.068 (2012).
25 Brito-Sanchez, Y. *et al.* Towards Better BBB Passage Prediction Using an Extensive and Curated Data Set. *Mol Inform* **34**, 308-330, doi:10.1002/minf.201400118 (2015).
26 Todarwal, Y. *et al.* Tau Protein Binding Modes in Alzheimer's Disease for Cationic Luminescent Ligands. *J Phys Chem B* **125**, 11628-11636, doi:10.1021/acs.jpcb.1c06019 (2021).
27 Murugan, N. A., Nordberg, A. & Agren, H. Cryptic Sites in Tau Fibrils Explain the Preferential Binding of the AV-1451 PET Tracer toward Alzheimer's Tauopathy. *ACS Chem Neurosci* **12**, 2437-2447, doi:10.1021/acschemneuro.0c00340 (2021).
28 Seidler, P. M. *et al.* Structure-based discovery of small molecules that disaggregate Alzheimer's disease tissue derived tau fibrils in vitro. *Nat Commun* **13**, 5451, doi:10.1038/s41467-022-32951-4 (2022).
