# Supplementary material for: Quantitative live cell imaging of a tauopathy model enables the identification of a polypharmacological drug candidate that restores physiological microtubule interaction
Source: Nat Commun. 2024 Feb 23;15:1679. doi: 10.1038/s41467-024-45851-6 (PMC10891143; doi:10.1038/s41467-024-45851-6)
Supplement: Supplementary file 3 — Reporting Summary [file 41467_2024_45851_MOESM3_ESM.pdf]

nature portfolio

Corresponding author(s): Roland Brandt

Last updated by author(s): Jan 25, 2024

Reporting Summary

Nature Portfolio wishes to improve the reproducibility of the work that we publish. This form provides structure for consistency and transparency in reporting. For further information on Nature Portfolio policies, see our Editorial Policies and the Editorial Policy Checklist.

Statistics

For all statistical analyses, confirm that the following items are present in the figure legend, table legend, main text, or Methods section.

n/a	Confirmed	
☐	☒	The exact sample size (*n*) for each experimental group/condition, given as a discrete number and unit of measurement
☐	☒	A statement on whether measurements were taken from distinct samples or whether the same sample was measured repeatedly
☐	☒	The statistical test(s) used AND whether they are one- or two-sided<br>*Only common tests should be described solely by name; describe more complex techniques in the Methods section.*
☒	☐	A description of all covariates tested
☐	☒	A description of any assumptions or corrections, such as tests of normality and adjustment for multiple comparisons
☐	☒	A full description of the statistical parameters including central tendency (e.g. means) or other basic estimates (e.g. regression coefficient) AND variation (e.g. standard deviation) or associated estimates of uncertainty (e.g. confidence intervals)
☐	☒	For null hypothesis testing, the test statistic (e.g. *F*, *t*, *r*) with confidence intervals, effect sizes, degrees of freedom and *P* value noted<br>*Give P values as exact values whenever suitable.*
☐	☒	For Bayesian analysis, information on the choice of priors and Markov chain Monte Carlo settings
☐	☒	For hierarchical and complex designs, identification of the appropriate level for tests and full reporting of outcomes
☒	☐	Estimates of effect sizes (e.g. Cohen's *d*, Pearson's *r*), indicating how they were calculated

Our web collection on statistics for biologists contains articles on many of the points above.

Software and code

Policy information about availability of computer code

Data collection	NIS-Elements VERSION AR 5.02.03 (Nikon, Japan)<br>Electron microscopy was performed on a Zeiss 10CR electron microscope<br>WB protein bands were detected by enhanced chemiluminescence with SuperSignal West Dura. Machine used Vilber Lourmat, Collégien, France.<br>Mass spectrometry data were collected by Q Exactive-Plus mass spectrometer (Thermo).<br>Ligand-based and structure-based analyses: in-house bash scripts
Data analysis	GraphPad Prism v8.0.1 (GraphPad Software, USA); Scripts used for FDAP analysis as described in Weissmann et al. (2009) are available at https://github.com/Department-of-Neurobiology/<br>Ligand-based and structure-based analyses: in-house bash scripts, grace, PLUMED

For manuscripts utilizing custom algorithms or software that are central to the research but not yet described in published literature, software must be made available to editors and reviewers. We strongly encourage code deposition in a community repository (e.g. GitHub). See the Nature Portfolio guidelines for submitting code & software for further information.

Data

Policy information about availability of data

All manuscripts must include a data availability statement. This statement should provide the following information, where applicable:

- Accession codes, unique identifiers, or web links for publicly available datasets
- A description of any restrictions on data availability
- For clinical datasets or third party data, please ensure that the statement adheres to our policy

Source data are provided with this paper. The proteomics data have been deposited in PRIDE.

Research involving human participants, their data, or biological material

Policy information about studies with human participants or human data. See also policy information about sex, gender (identity/presentation), and sexual orientation and race, ethnicity and racism.

Reporting on sex and gender	N/A
Reporting on race, ethnicity, or other socially relevant groupings	N/A
Population characteristics	N/A
Recruitment	N/A
Ethics oversight	N/A

Note that full information on the approval of the study protocol must also be provided in the manuscript.

Field-specific reporting

Please select the one below that is the best fit for your research. If you are not sure, read the appropriate sections before making your selection.

☒ Life sciences ☐ Behavioural & social sciences ☐ Ecological, evolutionary & environmental sciences

For a reference copy of the document with all sections, see nature.com/documents/nr-reporting-summary-flat.pdf

Life sciences study design

All studies must disclose on these points even when the disclosure is negative.

Sample size	The sample size for live-cell imaging experiments was chosen to meet the requirements to invoke the Central Limit Theorem. The central limit theorem, or a sample size which is large enough > 15 ensures that statistical tests applied (such as the t test and ANOVA) work well even when the populations are not perfectly Gaussian.<br>However, all data sets were tested for normality and transformed accordingly if necessary as described in the “Statistical Analysis” section.
Data exclusions	Samples of Live-cell imaging experiments were excledud by pre-established criteria, for details see Methods section "Live-cell imaging and Fluorescence Decay After Photoactivation (FDAP)" and the “Statistical Analysis”.
Replication	The data were obtained from independent experiments as described in the Figure legends. In addition, experiments were performed and statistically evaluated by different scientists.
Randomization	The allocation of samples into experimental groups was random.
Blinding	Experiments and statistical evaluation were performed by different scientists.

Reporting for specific materials, systems and methods

We require information from authors about some types of materials, experimental systems and methods used in many studies. Here, indicate whether each material, system or method listed is relevant to your study. If you are not sure if a list item applies to your research, read the appropriate section before selecting a response.

Materials & experimental systems

n/a	Involved in the study
☐	☒ Antibodies
☐	☒ Eukaryotic cell lines
☒	☐ Palaeontology and archaeology
☐	☒ Animals and other organisms
☒	☐ Clinical data
☒	☐ Dual use research of concern
☒	☐ Plants

Methods

n/a	Involved in the study
☒	☐ ChIP-seq
☒	☐ Flow cytometry
☒	☐ MRI-based neuroimaging

Antibodies

Antibodies used	PHF1 antibody (monoclonal mouse IgG1, kind gift of Peter Davies, Albert Einstein College of Medicine, New York, USA); peroxidase-conjugated AffiniPure donkey anti-mouse IgG (H+L) secondary antibody (Jackson ImmunoResearch Laboratories, Code number 715-035-151)
Validation	PHF1 antibody: Greenberg et al. (1992) J. Biol. Chem. 267:564-9.

Eukaryotic cell lines

Policy information about cell lines and Sex and Gender in Research

Cell line source(s)	PC12 cells (male), originally obtained from J.A. Wagner, Harvard Medical School
Authentication	Cells were authenticated by “Staatliches Gewerbeaufsichtsamt Hildesheim, Zentrale Unterstützungsstelle Abfall, Gentechnik und Gerätesicherheit” by STR profiling.
Mycoplasma contamination	The cell line had been negatively tested for mycoplasma contamination
Commonly misidentified lines (See ICLAC register)	*Name any commonly misidentified cell lines used in the study and provide a rationale for their use.*

Animals and other research organisms

Policy information about studies involving animals; ARRIVE guidelines recommended for reporting animal research, and Sex and Gender in Research

Laboratory animals	Primary cells from adult C57BL/6J mice, 3 months and 1 year old. C57BL/6J mice were kept and killed in accordance with the German animal care regulations based on the FELASA guidelines. The housing rooms were kept at a 12:12 light-dark cycle with a constant room temperature of 22 ± 2C and relative humidity of 55 ± 10%.
Wild animals	no wild animals were used in the study
Reporting on sex	Primary cells were prepared from male C57BL/6J mice
Field-collected samples	No field-collected samples were used in the study
Ethics oversight	Mice were kept and killed in accordance with the German animal care regulations based on the FELASA guidelines

Note that full information on the approval of the study protocol must also be provided in the manuscript.

Plants

Seed stocks	*Report on the source of all seed stocks or other plant material used. If applicable, state the seed stock centre and catalogue number. If plant specimens were collected from the field, describe the collection location, date and sampling procedures.*
Novel plant genotypes	*Describe the methods by which all novel plant genotypes were produced. This includes those generated by transgenic approaches, gene editing, chemical/radiation-based mutagenesis and hybridization. For transgenic lines, describe the transformation method, the number of independent lines analyzed and the generation upon which experiments were performed. For gene-edited lines, describe the editor used, the endogenous sequence targeted for editing, the targeting guide RNA sequence (if applicable) and how the editor was applied.*
Authentication	*Describe any authentication procedures for each seed stock used or novel genotype generated. Describe any experiments used to assess the effect of a mutation and, where applicable, how potential secondary effects (e.g. second site T-DNA insertions, mosiacism, off-target gene editing) were examined.*
